# Growth Cone Phosphoproteomics Reveals that GAP-43 Phosphorylated by JNK Is a Marker of Axon Growth and Regeneration

**DOI:** 10.1016/j.isci.2018.05.019

**Published:** 2018-05-31

**Authors:** Asami Kawasaki, Masayasu Okada, Atsushi Tamada, Shujiro Okuda, Motohiro Nozumi, Yasuyuki Ito, Daiki Kobayashi, Tokiwa Yamasaki, Ryo Yokoyama, Takeshi Shibata, Hiroshi Nishina, Yutaka Yoshida, Yukihiko Fujii, Kosei Takeuchi, Michihiro Igarashi

**Affiliations:** 1 Department of Neurochemistry and Molecular Cell Biology, Graduate School of Medical and Dental Sciences, Niigata University, 1-757 Asahimachi, Chuo-ku, Niigata 951-8510, Japan; 2 Center for Trans-disciplinary Research, Institute for Research Promotion, Niigata University, Chuo-ku, Niigata 951-8510, Japan; 3 Department of Neurosurgery, Brain Research Institute, Niigata University, Chuo-ku, Niigata 951-8585, Japan; 4 Precursory Research for Embryonic Science and Technology, Japan Science and Technology Agency, Kawaguchi, Saitama 332-0012, Japan; 5 Laboratory of Bioinformatics, Graduate School of Medical and Dental Sciences, Niigata University, Chuo-ku, Niigata 951-8510, Japan; 6 Department of Developmental and Regenerative Biology, Medical Research Institute, Tokyo Medical and Dental University, Bunkyo-ku, Tokyo 113-8510, Japan; 7 K.K. Sciex Japan, Shinagawa-ku, Tokyo 140-0001, Japan; 8 Center for Coordination of Research, Institute for Research Promotion, Niigata University, Ikarashi, Niigata 951-2181, Japan; 9 Department of Medical Cell Biology, Aichi Medical University, Nagakute, Aichi 480-1195, Japan

**Keywords:** Neuroscience, Developmental Neuroscience, Bioinformatics, Proteomics

## Abstract

Neuronal growth cones are essential for nerve growth and regeneration, as well as for the formation and rearrangement of the neural network. To elucidate phosphorylation-dependent signaling pathways and establish useful molecular markers for axon growth and regeneration, we performed a phosphoproteomics study of mammalian growth cones, which identified >30,000 phosphopeptides of ∼1,200 proteins. The phosphorylation sites were highly proline directed and primarily MAPK dependent, owing to the activation of JNK, suggesting that proteins that undergo proline-directed phosphorylation mediate nerve growth in the mammalian brain. Bioinformatics analysis revealed that phosphoproteins were enriched in microtubules and the cortical cytoskeleton. The most frequently phosphorylated site was S96 of GAP-43 (growth-associated protein 43-kDa), a vertebrate-specific protein involved in axon growth. This previously uncharacterized phosphorylation site was JNK dependent. S96 phosphorylation was specifically detected in growing and regenerating axons as the most frequent target of JNK signaling; thus it represents a promising new molecular marker for mammalian axonal growth and regeneration.

## Highlights

• Phosphoproteomics of mammalian growth cone membranes reveals activation of MAPK
• JNK is the activated MAPK in growth cones and phosphorylates S96 of GAP-43
• pS96 of GAP-43, the most frequent site, is observed in growing axons
• pS96 is biochemically detected in the regenerating axons of the peripheral nerves

## Graphical Abstract

**Figure:**
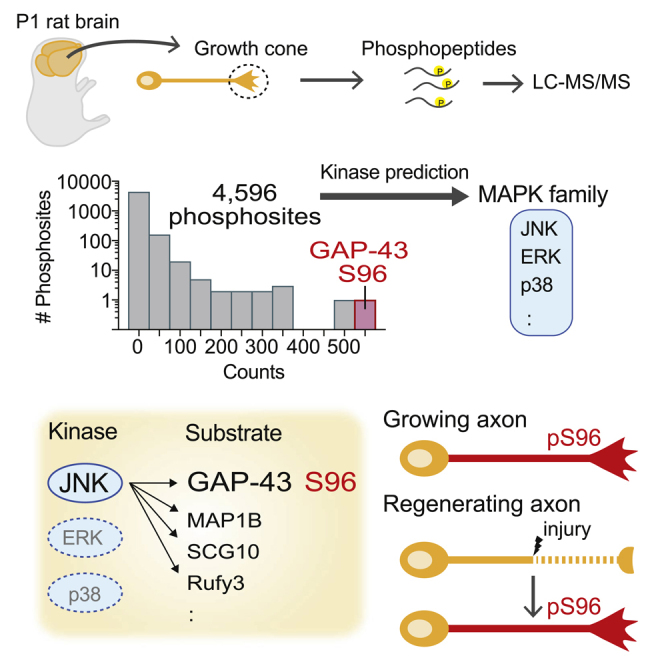

## Introduction

The growth cone, a specialized, highly motile structure formed at the tips of extending axons of developing neurons (Dent et al., 2011, Igarashi, 2014), is crucial for accurate synaptogenesis in the developing brain. In addition, growth cone activity is involved in the rearrangement of neuronal networks during neural plasticity and axonal regeneration in the adult brain (Bloom and Morgan, 2011, Gordon-Weeks and Fournier, 2014, Nozumi and Igarashi, 2017, Tamada and Igarashi, 2017). Therefore, to understand the mechanisms underlying neuronal network formation and maintenance, it is essential to elucidate the molecular pathways that determine growth cone behavior. At present, however, little molecular information is available regarding growth cones in the mammalian brain. Previously, we performed a proteomics analysis of mammalian growth cones and characterized approximately 1,000 unique proteins (Nozumi et al., 2009; see also Estrada-Bernal et al., 2012). The results of this analysis revealed novel molecular mechanisms underlying nerve growth (Igarashi, 2014, Nozumi et al., 2017, Honda et al., 2017a, Honda et al., 2017b).

To further investigate molecular signaling in growth cones, we focused on protein phosphorylation, the most important regulatory mechanism in many cellular processes (Humphrey et al., 2015a). To date, most efforts in this regard have used *in vitro* phosphorylation systems that do not necessarily represent the *in vivo* situation. Phosphoproteomics is an important, novel, and powerful technique for comprehensive and quantitative identification of *in vivo* phosphorylation sites (von Stechow et al., 2015) and should be able to establish novel molecular markers for axonal growth and regeneration.

Specifically, we performed phosphoproteomics analysis of the growth cone membrane (GCM; Ellis et al., 1985, Nozumi et al., 2009, Igarashi, 2014). From among more than 30,000 phosphopeptides, this analysis identified ∼4,600 different phosphorylation sites from ∼1,200 proteins. Surprisingly, proline (P)-directed phosphorylation was predominant, with more than 60% of serine (S) or threonine (T) phosphorylation sites predicted to depend on P-directed kinases. Bioinformatics analysis suggested that these frequent P-directed phosphorylation events were due to mitogen-activated protein kinase (MAPK) activation. In particular, we found that c-Jun *N*-terminus kinase (JNK; Bogoyevitch et al., 2010) was the major active member of the MAPK family and was responsible for several heavily phosphorylated sites.

The most abundant phosphorylated site was S96 of *neuronal growth-associated 43-kDa* (GAP-43, also called as neuromodulin), a vertebrate neuron-specific protein involved in nerve growth (Skene, 1989, Denny, 2006, Holahan, 2017), comprising more than 1% of all phosphopeptides. This phosphorylated site was previously uncharacterized. Subsequent experiments revealed that S96 phosphorylation (pS96) was JNK dependent. A pS96 antibody (Ab) specifically recognized growing and regenerating axons, and pS96 was directly detected in regenerating axons by mass spectrometry (MS).

Taken together, our data show that JNK signaling is a key pathway for axon growth that is conserved across a wide range of animals. JNK signaling via vertebrate-specific substrates such as GAP-43 plays important roles in mammalian growth cones, and pS96 Ab represents a promising new molecular marker for mammalian axonal growth/regeneration.

## Results

### High Frequency of P-Directed Phosphosites in GCMs

Phosphoproteomics analysis of GCM fractions isolated from postnatal day 1 (P1) rat forebrain identified more than 30,000 phosphopeptides at greater than 95% confidence (see Data S1). The condensation ratio of the phosphopeptides (i.e., the ratio of phosphopeptides to total peptides) was 95.9%. Thresholding with 1% false discovery rate (FDR) extracted 4,596 phosphorylation sites that corresponded to 1,223 proteins. Highly frequent phosphorylation sites are shown in Table S1.

**Figure 1:**
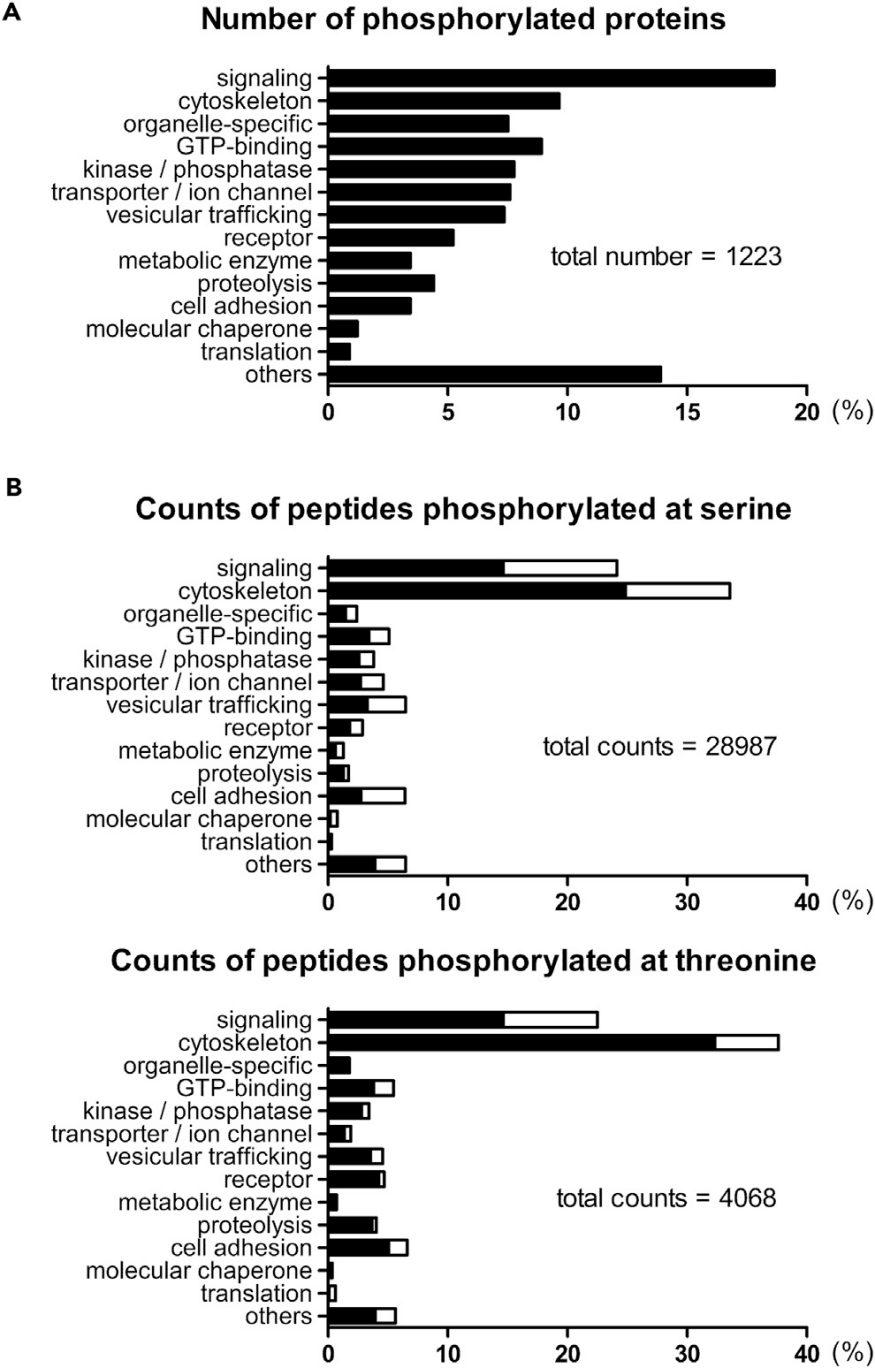
GCM Phosphopeptides Derived from P1 Rat Brain Reveal a Large Number of P-Directed Kinase Substrates (A) Classification of phosphoproteins (1,223 proteins in total) that were derived from the phosphopeptides (4,596 species) detected by MS with 1% FDR. The value in each row represents the fraction of proteins in each functional category. (B) Counts of peptides phosphorylated at serine (28,987 total counts) and threonine (4,068 total counts) that belong to each protein category. The counts were further divided into those for P-directed sites (*filled bars*) and those for non-P-directed sites (*open bars*).

**Figure 2:**
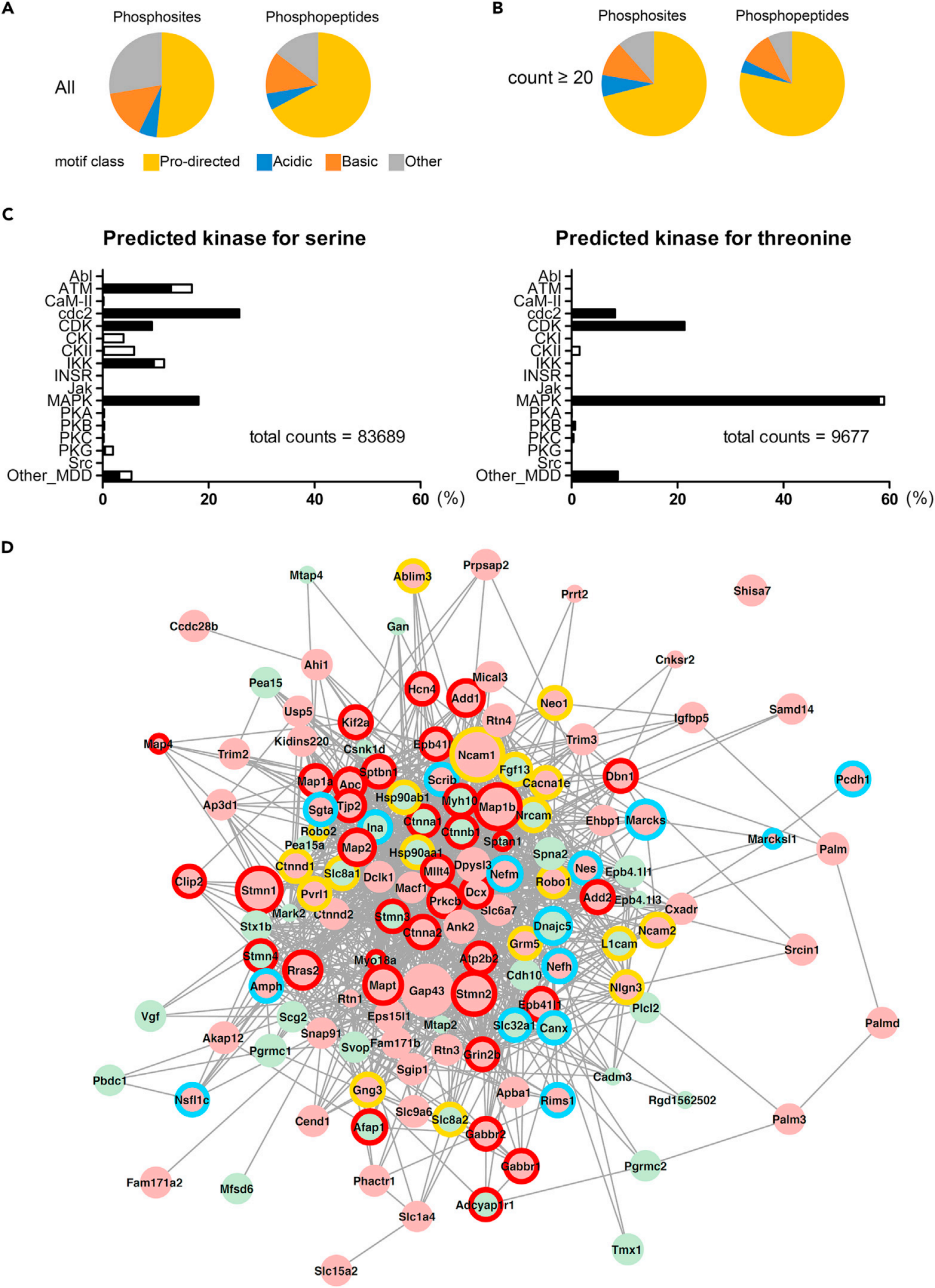
Bioinformatics Analysis of Phosphosites Identified by GCM Phosphoproteomics of Rat P1 Brain Reveals that P-Directed Sites Are Mainly Dependent on MAPK (A and B) Fractions of phosphosites (*left*) and phosphopeptides (*right*) that are substrates of acid, basic, and proline-directed kinases for all data (A) or data thresholded by 20 counts (B). (C) Protein kinases predicted for the serine (*left*) and threonine (*right*) phosphosites using KinasePhos server. The value in each row represents the fraction of phosphopeptides that are the targets of each kinase. The fractions were further divided into P-directed (*filled bars*) or non-P-directed (*open bars*) phosphorylation. (D) Protein association network for P-directed and non-P-directed proteins. Protein association network was constructed using the STRING database (Szklarczyk et al., 2017), merged with data from human, rat, and mouse. *Red* and *green* filled circles indicate P-directed and non-P-directed phosphorylated proteins, respectively. The size of the circle for each protein represents its phosphorylation frequency in GCM. The colors of the external rings indicate enriched protein network groups: group I (*red*), cytoskeletal proteins (microtubule-related proteins, cortical skeletal proteins, and actin-binding proteins); group II (*yellow*), signaling molecules related to axon growth/guidance (cell adhesion molecules, proteins in cAMP- or Ca^2+^-dependent signaling pathways, small GTPase signaling molecules, and guidance receptors); and group III (*blue*), other categories. Proteins without the external rings were not enriched.

We classified the kinase substrates in GCMs into various categories based on the number of phosphorylation sites (Figure 1A) and the frequency of phosphopeptides phosphorylated at S or T (Figure 1B). Cytoskeletal components and signaling proteins were the major GCM phosphoproteins identified in this manner (Figures 1A and 1B; see also Data S2, referring to the protein names). Among the phosphopeptides identified in GCMs, serine-proline (SP)/threonine-proline (TP) residues, i.e., P-directed-kinase-dependent phosphorylation sites (Villén et al., 2007, Huttlin et al., 2010), were highly enriched in the GCM (Figures 1B, 2A, and 2B; Table S1).

Protein kinases are classified into four major groups: acidic, basic, P directed, and others (Villén et al., 2007). P-directed sites constituted 63.9% of phosphoserine (pS) and 78.0% of phosphothreonine (pT) sites in all categories (Figure 1B; Data S1). The typical sequences of each protein kinase category were visualized using the *IceLogo* web server (Figure S1). The fraction of P-directed sites (Figures 2A and 2B) was higher than those estimated from a meta-analysis of two previous reports on phosphoproteomics (Lundby et al., 2013, Humphrey et al., 2015b; Figure S2).

Next, we predicted kinases that are responsible for the phosphorylation sites identified by our analysis. Using a kinase-specific phosphorylation site prediction tool KinasePhos (Huang et al., 2005, Wong et al., 2007), we found that MAPK is most likely to be a kinase responsible for the phosphorylation of SP/TP sites with high frequencies (Figure 2C). To elucidate the physiological functions of these substrates, we performed enrichment analysis using the GCM phosphorylation data, particularly for phosphopeptides that were phosphorylated ≥20 times (Figure 2B; Data S3). Two groups containing such highly phosphorylated sites, cytoskeleton-associated proteins (group I) and signaling proteins including cell adhesion molecules and guidance receptors (group II), were also highly enriched in the protein networks (Figure 2D). Substrates with P-directed phosphorylated sites (Figure 2C) were also enriched (Figure 2D). These proteins are thought to be involved in axon growth and guidance in mammalian brain (Dent et al., 2011, Igarashi, 2014, Short et al., 2016, Batty et al., 2017). Therefore, our results suggest that highly concentrated P-directed phosphoproteins in GCM play important functional roles in mammalian axon growth and guidance.

### P-Directed Phosphorylation of GCM Phosphoproteins Requires JNK Activity

The MAPK family includes extracellular-signal-regulated kinase (ERK), p38, and JNK, among which JNK appeared to be the most likely kinase candidate for mammalian GCM phosphorylation. First, several recent reports showed that JNK is involved in multiple steps of mammalian brain development (Oliva et al., 2006, Hirai et al., 2011, Barnat et al., 2010, Coffey, 2014). Second, JNK signaling is activated during axon regeneration, even in *C. elegans* (Nix et al., 2011). Together, these observations suggest the importance of JNK signaling in a wide range of organisms.

**Figure 3:**
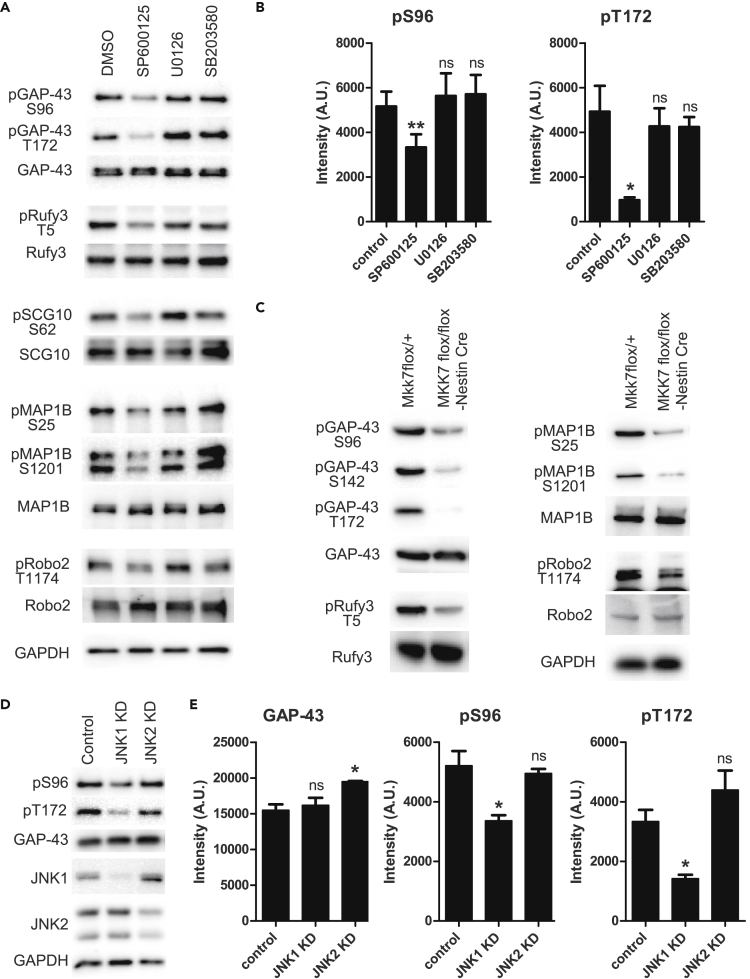
MAPK Substrates Identified by GCM Phosphoproteomics Undergo JNK-Dependent Phosphorylation (A and B) Mouse cortical neurons were treated with 20 μM SP600125 (JNK inhibitor), 5 μM U0126 (MEK1/2 inhibitor), or 5 μM SB203580 (p38 inhibitor) for 3 hr. As a control, an equal volume of the solvent DMSO was added to the medium. (A) The SP/TP phosphorylated sites of various GCM proteins are JNK dependent. Frequencies not appearing in Table S1 are as follows: Robo2 [pT1154] = 25; GAP-43 [pS142] = 18; and Rufy3 [pT5] = 19. Western blot results of non-phosphospecific Abs are shown as negative controls. Kinase inhibitors did not affect the reactivity of any of these non-phosphospecific Abs. (B) Effects of MAPK inhibitors on GAP-43 phosphorylation at S96 and T172. Values represent the measured intensity (mean ± SEM, n = 3). **p < 0.01; *p < 0.05; ns, p > 0.05. One-way repeated measures ANOVA with Bonferroni tests to the control. (C) Brain-specific cKO of MKK7 (Yamasaki et al., 2011), an upstream activator of JNK, suppressed the identified SP/TP phosphorylation. Brain extracts from WT and MKK7^flox/flox^ Nestin-Cre embryos at E15.5 were analyzed by immunoblotting using the indicated Abs. Western blot results of non-phosphospecific Abs are shown as the controls. Kinase inhibitors did not affect the reactivity of any of these non-phosphospecific Abs. (D and E) Effects of mouse JNK knock down on GAP-43 pS96 and pT172. The representative western blotting (D) and quantified (E) data. Values represent the measured intensity (mean ± SEM, n = 3). *p < 0.05; ns, p > 0.05. One-way ANOVA with Bonferroni tests to the control. *GAPDH: glyceroaldehyde-3-phosphate dehydrogenase* (A, C, and D).

To test this hypothesis, we produced eight phospho-specific Abs against the high-frequency SP/TP phosphorylation sites (total number = 1,163, corresponding to 3.8% of the Conf95 phosphopeptides; Figure 3A; see also Tables S1 and S2). The most frequently phosphorylated site was S96 of GAP-43. When we chemically inhibited MAPK family members, only the JNK inhibitor SP600125 specifically blocked phosphorylation of GAP-43, as determined using phospho-specific Abs (Figures 3A and S3A; the antigen sequences are shown in Table S2). The intensities of two sites in GAP-43, S96 and T172, were decreased by SP600125 treatment (Figure 3B).

We also identified the upstream signal that activates JNK. In the brain, MKK7 is a specific activator of JNK (Yamasaki et al., 2011), and mice with brain-specific conditional knockout (cKO) of MKK7 exhibit hypoactive axon formation in the developing brain (Yamasaki et al., 2011, Yamasaki et al., 2017). Using our phospho-specific Abs in this cKO mouse, we found that phosphorylation signals in the brain were greatly diminished, as also observed with the chemical inhibitor (Figure 3C). By contrast, cyclin-dependent kinase 5 (CDK5) and glycogen synthase kinase 3β (GSK3β) inhibitors did not prevent GAP-43 phosphorylation, suggesting that neither of these kinases is responsible for S96 or T172 phosphorylation (Figures S3B and S3C).

JNK has three isoforms: JNK1, JNK2, and JNK3 (Haeusgen et al., 2009, Coffey, 2014). Because JNK3 is not involved in early brain development (Kuan et al., 2003) and its expression is lower than that of the other two, we did not investigate its role in these experiments. Treatment of the cultured cortical neurons with small interfering RNAs (siRNAs) against JNK1 and JNK2 revealed that JNK1 plays a more important role in GAP-43 phosphorylation in mouse brain (Figures 3D and 3E), as expected (Hirai et al., 2011). JNK is activated in murine developing neurons (Chang et al., 2003). Taken together with our data, this observation suggests that JNK is responsible for the phosphorylation of many GCM proteins with SP/TP sites, such as S96 in GAP-43.

### Growing Axons Are Associated with pS96 GAP-43

To further investigate the biological significance of the JNK-dependent, highly P-directed phosphosites in mammalian nerve growth, we focused on pS96 of GAP-43, both because it was the most abundant (Table S1) and because GAP-43 is a classical molecular marker for vertebrate axon growth and regeneration. pS96 Ab recognized exogenously expressed wild-type GAP-43 but not a mutant (S96A) lacking the phosphorylation site, especially under hyperosmotic conditions (0.5 M NaCl), which activate JNK (Figures S4A and S4B) (Zhang and Cohen, 1996).

**Figure 4:**
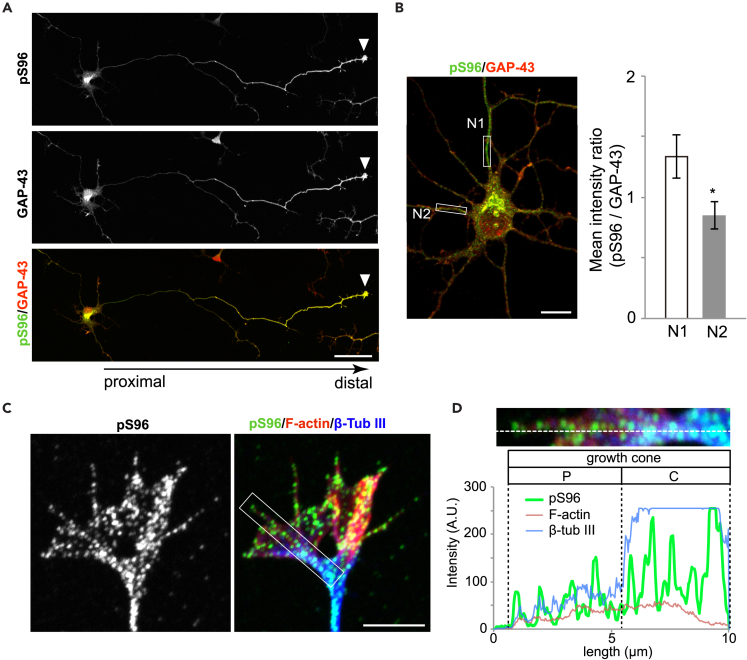
Antibody Specific for pS96 of GAP-43 Selectively Recognizes Developing and Renewing Axons (A and B) Immunostaining of cultured mouse hippocampal neurons (3 days of culture) using antibodies against pS96 (*green*) and total GAP-43 (*red*). (A) Full view of a single neuron with a long neurite and a growth cone (*arrowhead*). Scale bar: 50 μm. (B) Magnified view of a soma with several neurites. (*Left*) White boxes indicate regions of interest (ROI) that were measured. (*Right*) Means of the staining intensity ratios (pS96 vs pan-GAP-43) in neurites. *N1*: longest neurite; *N2*: second-longest neurite. Scale bar: 10 μm. Values are expressed as means ± SEM; n = 3; *p < 0.05. (C and D) Co-localization of pS96 (*green*) with F-actin (*red*) and β-tubulin III (*blue*) in the growth cone of mouse cortical neurons. Scale bar: 5 μm. (C) White box indicates the ROI that was measured. (D) Quantitative distribution of the ROI (C) by measuring the fluorescence intensity along the white dashed line from the filopodial tip. P: peripheral domain; C: central domain.

In cultured mouse neurons, this pS96 Ab intensely labeled distal axons and growth cones with a punctate labeling pattern that was particularly concentrated at the growth cone (Figure 4A). Labeling was much weaker in proximal axons (Figure 4A) and other minor processes (Figure 4B). pS96 Ab immunoreactivity in growth cones was distributed along filopodial actin filaments and microtubules in the central domain (Figures 4C and 4D).

**Figure 5:**
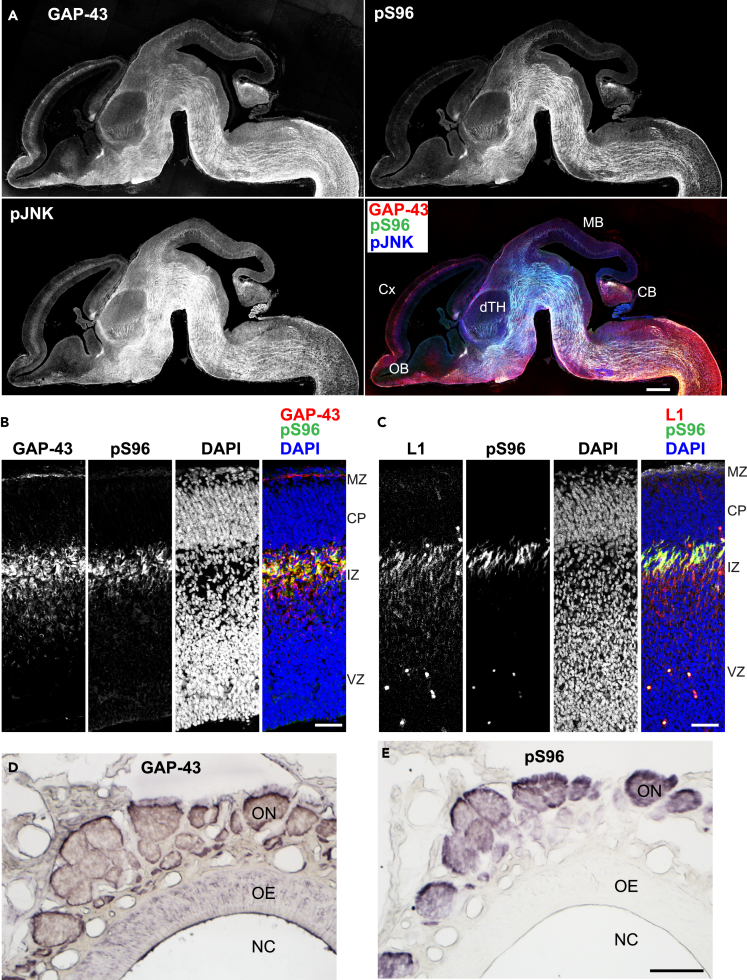
Expression Pattern of GAP-43, pS96, and pJNK in Developing Mouse Brain (A) Expression in an E15 parasagittal section stained with pan-GAP-43, pS96, and pJNK Abs. GAP-43 itself was expressed in most of the differentiated neurons; by contrast, pS96 was localized to axonal processes but was not detected in cell bodies. pJNK exhibited a broader distribution than GAP-43. OB, olfactory bulb; Cx, neocortex; dTH, dorsal thalamus; MB, midbrain; CB, cerebellum. (B and C) Expression pattern of pS96 and GAP-43 (B) or pS96 and the cell adhesion molecule L1 (C). Nuclear staining with DAPI is also shown. GAP-43 itself was expressed by migrating neurons and ingrowing axons in the intermediate zone (IZ). pS96 expression was restricted to the L1-positive thalamocortical axons in the upper IZ. MZ, marginal zone; CP, cortical plate; VZ, ventricular zone. (D and E) Expression in the primary olfactory system on P14. GAP-43 itself was expressed in the cells of the olfactory epithelium (OE) and the olfactory nerves (ON; D), whereas pS96 was localized only in the ON (E). NC, nasal cavity. Scale bars: 50 μm.

Immunohistochemical analyses revealed that pS96 Ab specifically recognized growing axons *in vivo* during development (Figures 5A–5C, S5A, and S5B). On embryonic day (E) 15, GAP-43 itself was expressed in most of the differentiated neurons, whereas by contrast, pS96 was localized to axonal processes but not present in cell bodies (Figure 5A). pJNK signals partially overlapped with those of pS96 (Figure 5A). GAP-43 was expressed in most of the differentiated neurons, but *in vivo* immunostaining revealed that pS96 was localized only to axonal processes, not to cell bodies (Figures 5 and S4C–S4F), although in culture, the cell bodies were stained by this Ab (Figure 4A). In samples co-stained for the cell adhesion molecule L1 and nuclear DNA (with DAPI), GAP-43 itself was expressed by migrating neurons and ingrowing axons in the intermediate zone (IZ), whereas pS96 was restricted to L1-positive thalamocortical axons in the upper IZ. Such axon-specific expression pattern of pS96 was widely distributed in various fiber tracts of the developing brain (Figure S6):

Adult mice contain continuously renewing olfactory axons, and GAP-43-positive cells represent new neurons derived from stem cells in the basal region of the epithelium (Margolis et al., 1991). We performed immunohistochemistry using the pS96 Ab to determine whether these regenerating, newly growing axons were stained like the growing axons of developing neurons (Figures 5D and 5E). In contrast to the conventional pan GAP-43 Ab, the pS96 Ab more precisely detected growing axons and more heavily immunostained nerve bundles and nerves exiting the olfactory epithelium but did not recognize cell bodies (Figures 5D and 5E).

Taken together, our data show that growing axons *in vivo* were invariably associated with JNK-dependent pS96 of GAP-43, indicating that this Ab represents a new specific molecular marker for growing axons that is superior to anti-pan-GAP-43 Ab.

### pS96 Is a Marker for Axon Regeneration in the Peripheral Nervous System

**Figure 6:**
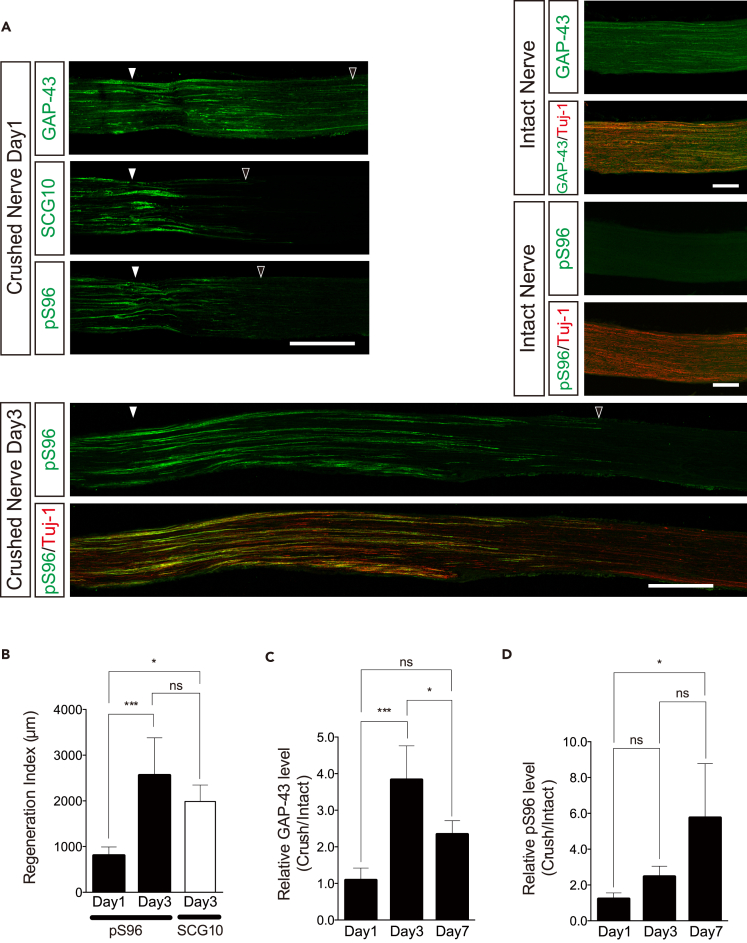
Axon Regeneration of Injured Sciatic Nerves in Adult Mice Is Strongly Associated with S96 Phosphorylation of GAP-43 (A) Immunohistochemistry of longitudinal sections of sciatic nerves at days 1 and 3 after crush injury using pS96, total GAP-43, SCG10, and Tuj-1(neuron-specific β3 tubulin) Abs. SCG10 was used as a positive control for axon regeneration. *Arrowheads* (*white*), injury point; (*black*), the farthest point of positive immunoreactivity. *Intact nerves* indicate immunohistochemistry of uninjured nerves. Note that pS96 Ab did not label *intact nerves*. Scale bars: 500 μm (*days 1 and 3*); 200 μm (*intact*). (B) Regeneration index (Shin et al., 2014) of pS96 (see Transparent Methods) was higher on day 3 than on day 1. n = 4 (day 1), and n = 6 (day 3); *p < 0.05, ***p < 0.001 by one-way *ANOVA* with Bonferroni tests. (C and D) Quantification of GAP-43 and pS96 on western blots incubated with their specific Abs. Both crushed sciatic nerves and contralateral, intact ones were excised and subjected to blotting using pan-GAP-43 and pS96 Abs on days 1, 3, and 7 after crushing (n = 4 for days 1, 3, and 7). The blot intensities of the proteins in the intact nerve were used as controls. *p < 0.05; ***p < 0.001 by one-way *ANOVA* with Bonferroni tests. All data in (B–D) are expressed as means ± SD.

Peripheral nervous system (PNS) axons in mammals, including humans, can regenerate (Doron-Mandel et al., 2015). In light of the findings described earlier, we asked whether regenerating PNS axons are associated with pS96 of GAP-43. To answer this question, we generated an injury model of the sciatic nerve in mice. In all the mice used for this regeneration analysis, we confirmed the “Sciatic Functional Index for Mouse” (Navarro, 2015) before and after the injury (Savastano et al., 2014). Three days after the nerve crush, we clearly observed pS96 Ab immunoreactivity (Figure 6A), suggesting that the pS96-positive axons elongate with time and that they correspond to regenerating neurons after injury. To quantify the regeneration, we calculated the regeneration index, which is the distance from the injury site to the point where the signal intensity drops by half (Shin et al., 2014). The index for pS96 significantly increased on day 3, consistent with that for SCG10 (Stmn2), another marker of axon regeneration (Figure 6B; Shin et al., 2014). pS96 signals remained low in the intact nerve (Figure 6A). The “crush” vs “intact” ratio of pS96 signal intensity continuously increased until day 7 (Figure 6C), whereas that of GAP-43 transiently increased on day 3 and then decreased on day 7 (Figure 6C), suggesting that pS96 captures axon regeneration more faithfully than GAP-43 itself.

### Detection of pS96 in Regenerating Axons by Phosphoproteomics of Single Injured Sciatic Nerves

**Figure 7:**
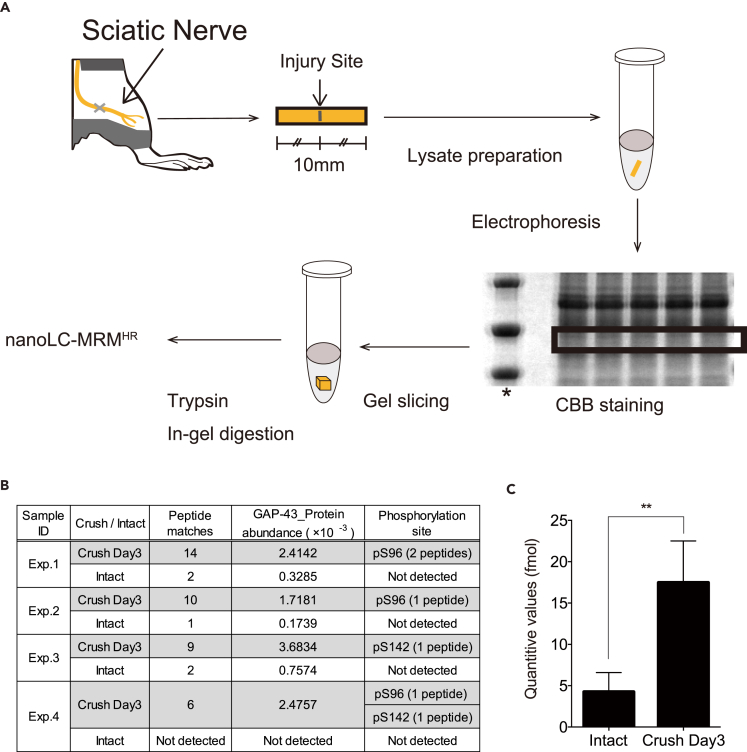
Phosphoproteomics of a Single, Injured Sciatic Nerve in Adult Mice Reveals that pS96 Is Specifically Detected at Regenerating Axons (A) Schematic of phosphoproteomics procedure for a single injured sciatic nerve. The crushed region of a sciatic nerve was excised and divided into 1 cm segments, which were prepared for SDS-PAGE. The band corresponding to GAP-43 was cut out, trypsinized in-gel, and subjected to MS analysis. *, molecular mass marker. (B) Shotgun phosphoproteomics analysis of a single crushed nerve. Note that pS96 was detected in three of five injured samples, and another JNK-dependent site, pS142 (Figure 3C), was also detected twice. By contrast, no phosphorylated peptides of GAP-43 were detected in intact nerves. (C) MS quantification of pS96 using HR-MRM to compare crushed (*Crush*) and intact sciatic nerves on day 3 after injury. *Crush* represents the regenerating axons. **p < 0.01 (Student's t test). All data are shown as means ± SD.

Next, we tried to detect pS96 by MS. For this purpose, samples likely to include regenerating axons were excised from a single sciatic nerve 3 days after injury. After lysis and electrophoresis (Figure 7A), the region of the SDS-PAGE gel corresponding to the position of GAP-43 was cut out and analyzed by liquid chromatography (LC)-MS. This approach positively detected pS96 of GAP-43, and in some cases pS142 (see Figure 3C), another P-directed phosphorylation site of GAP-43 (Figure 7B). These analyses with small samples sensitively and specifically detected pS96 of GAP-43 in regenerating axons, but not in undamaged axons (Figure 7B), consistent with the immunohistochemical results (Figure 6A). By contrast, we could not detect protein kinase C (PKC)-dependent S41 phosphorylation, which had been classically focused on by *in vitro* phosphorylation studies (Skene, 1989, Apel et al., 1990, Denny, 2006) (Figure 7B). Using high-resolution (HR) multiple-reaction monitoring (MRM) for quantification of specific sets of proteins in phosphoproteomics (Figure S7), we confirmed that the level of pS96 GAP-43 was more than 4-fold higher in regenerating axons than in intact nerves (Figure 7C).

These results indicate that pS96 of GAP-43 is tightly associated with PNS axon regeneration and involved in the functional recovery that accompanies regeneration. In addition, these findings confirm that pS96 Ab is a promising molecular marker for regenerating axons, as well as those growing during normal development.

## Discussion

In this study, we performed quantitative profiling of phosphoproteins and their phosphorylation sites in mammalian GCMs. We obtained three important results: (1) The high frequency of P-directed phosphorylation in GCMs (Figures 2A and 2B; Table S1), as revealed by bioinformatics and biological experiments, was primarily due to MAPKs (Figure 2C), and in particular JNK (Figure 3). (2) The most abundant phosphorylated site, S96 of GAP-43 (Table S1), also underwent JNK-dependent phosphorylation (Figure 3). In addition (3), pS96 was tightly associated with both developmental axon growth (Figures 4, 5, S5, and S6) and axon regeneration (Figures 6 and 7).

Our results suggest that mammalian nerve growth requires activation of JNK. Surprisingly, this conclusion is essentially consistent with the requirement for JNK signaling in axon regeneration, consistent with the results of a large number of mutant screens in *C. elegans* (Hammarlund et al., 2009, Yan et al., 2009, Chen et al., 2011, Andrusiak and Jin, 2016). We conclude that JNK signaling is evolutionarily conserved, even in mammalian axon growth, as demonstrated by the large number of substrates that depend on P-directed kinases. Although S96 phosphorylation was discovered using classical methodology more than a quarter century ago (Spencer et al., 1992), this was before the discovery of JNK (Hibi et al., 1993); however, there have been no reports showing a link between pS96 and JNK to date. We also demonstrated that JNK supports axon growth by modulating vertebrate-specific substrates such as GAP-43 (Figure 3) during mammalian development (Holahan, 2017). Although pS96 was found within the supplemental large datasets of the adult mouse brain phosphoproteomics (Huttlin et al., 2010, Lundby et al., 2012), its functions in adult brain is not known.

Our results show that pS96 of GAP-43 is associated with normal mammalian axon growth (Figures 4 and 5) and regeneration (Figures 6 and 7), probably because it is the best substrate for JNK (Table S1). Therefore, the pS96 Ab represents a promising marker for growing and regenerating axons in rodents. Previously, we reported that pS96 Ab could be involved in the regeneration of nerves after surgery (Oyamatsu et al., 2012); however, at that time, the importance of pS96 was not clearly understood. Here, the proteomic results (Figures 2 and 3; Table S1) provide a background against which to understand the importance of this phosphorylation. In addition, pS96 could be detected by MS at high levels, even in a single regenerating segment of injured PNS axons (Figures 7B and 7C). Considering that GAP-43 itself was widely distributed throughout the cell bodies of the developing neurons (Figures 5A–5C) and was present in the intact mature neurons (Figure 6A), pS96 could be used as a strong and specific marker for axon growth and regeneration in rodents. We recently succeeded in performing super-resolution microscopy of the behaviors of live growth cones, revealing new endocytic mechanisms for nerve growth (Nozumi et al., 2017, Nozumi and Igarashi, 2017). This new method should help reveal the function of pS96 signaling in growth cone behavior much more precisely.

It is important to note that JNK is also a negative regulator of axon growth and can induce axon degeneration (Miller et al., 2009, Tedeschi and Bradke, 2013, Lu et al., 2014, Yang et al., 2015). Accordingly, based on all available data, we conclude that JNK physiologically contributes to axon growth (Yamasaki et al., 2012). The ability to control JNK activity in the near future may lead to effective axon regeneration, enabling clinical treatment of intractable neurological diseases and neural injuries.

## Methods

All methods can be found in the accompanying Transparent Methods supplemental file.

## Supplemental Information

Document S1. Transparent Methods, Figures S1–S7, and Tables S1–S5

Data S1. Phosphoproteomics of the GCM Proteins, Related to Figure 1 A: Time; B: Precise molecular weight; C: Precise m/z; D: Precise charge (z); E: Protein number (see Data S2); F: Best sequence; G: Modification; H: Confidence; I: Theoretical mass; J: Charge (z).

Data S2. Identified GCM Proteins Analyzed with Phosphoproteomics, Related to Figure 1 A: Protein number (see Data S1); B: Unused; C: Total; D: % coverage; E: Accession number; F: Protein name. *Yellow line*: no phosphorylation-modified peptides were found (only other modifications such as methylation, etc.); *Red line:* no phosphopeptides with >95% reliability were found. They have been deleted from the total counts (Figure 1).

Data S3. Enrichment Analysis of the GCM Phosphoproteins, Related to Figure 2 *Category*, KEGG BRITE categories were used for enrichment analysis. The number of enriched genes was counted in the fourth level of the categories. If the number of genes included in a category is lower than four, the category is removed from the calculation. p*-value*, p-value of Fisher's exact test; *Q-value*, FDR adjusted p-value; *Genes*, the genes encoding the identified phosphoproteins in GCM. See also Figure 2D.

## References

[bib1] Andrusiak M.G., Jin Y. (2016). Context specificity of stress-activated mitogen-activated protein (MAP) kinase signaling: the story as told by *Caenorhabditis elegans*. J. Biol. Chem..

[bib2] Apel E.D., Byford M.F., Au D., Walsh K.A., Storm D.R. (1990). Identification of the protein kinase C phosphorylation site in neuromodulin. Biochemistry.

[bib3] Barnat M., Enslen H., Propst F., Davis R.J., Soares S., Nothias F. (2010). Distinct roles of c-Jun *N*-terminal kinase isoforms in neurite initiation and elongation during axonal regeneration. J. Neurosci..

[bib4] Batty N.J., Fenrich K.K., Fouad K. (2017). The role of cAMP and its downstream targets in neurite growth in the adult nervous system. Neurosci. Lett..

[bib5] Bloom O.E., Morgan J.R. (2011). Membrane trafficking events underlying axon repair, growth, and regeneration. Mol. Cell Neurosci..

[bib6] Bogoyevitch M.A., Ngoei K.R., Zhao T.T., Yeap Y.Y., Ng D.C. (2010). c-Jun *N*-terminal kinase (JNK) signaling: recent advances and challenges. Biochim. Biophys. Acta.

[bib7] Chang L., Jones Y., Ellisman M.H., Goldstein L.S., Karin M. (2003). JNK1 is required for maintenance of neuronal microtubules and controls phosphorylation of microtubule-associated proteins. Dev. Cell.

[bib8] Chen L., Wang Z., Ghosh-Roy A., Hubert T., Yan D., O'Rourke S., Bowerman B., Wu Z., Jin Y., Chisholm A.D. (2011). Axon regeneration pathways identified by systematic genetic screening in *C. elegans*. Neuron.

[bib9] Coffey E.T. (2014). Nuclear and cytosolic JNK signalling in neurons. Nat. Rev. Neurosci..

[bib11] Denny J.B. (2006). Molecular mechanisms, biological actions, and neuropharmacology of the growth-associated protein GAP-43. Curr. Neuropharmacol..

[bib12] Dent E.W., Gupton S.L., Gertler F.B. (2011). The growth cone cytoskeleton in axon outgrowth and guidance. Cold Spring Harbor Perspect. Biol.

[bib13] Doron-Mandel E., Fainzilber M., Terenzio M. (2015). Growth control mechanisms in neuronal regeneration. FEBS Lett..

[bib59] Ellis L., Wallis I., Abreu E., Pfenninger K.H. (1985). Nerve growth cones isolated from fetal rat brain. IV. Preparation of a membrane subfraction and identification of a membrane glycoprotein expressed on sprouting neurons. J. Cell Biol..

[bib14] Estrada-Bernal A., Sanford S.D., Sosa L.J., Simon G.C., Hansen K.C., Pfenninger K.H. (2012). Functional complexity of the axonal growth cone: a proteomic analysis. PLoS One.

[bib17] Gordon-Weeks P.R., Fournier A.E. (2014). Neuronal cytoskeleton in synaptic plasticity and regeneration. J. Neurochem..

[bib18] Haeusgen W., Boehm R., Zhao Y., Herdegen T., Waetzig V. (2009). Specific activities of individual c-Jun *N*-terminal kinases in the brain. Neuroscience.

[bib19] Hammarlund M., Nix P., Hauth L., Jorgensen E.M., Bastiani M. (2009). Axon regeneration requires a conserved MAP kinase pathway. Science.

[bib20] Hibi M., Lin A., Smeal T., Minden A., Karin M. (1993). Identification of an oncoprotein- and UV-responsive protein kinase that binds and potentiates the c-Jun activation domain. Genes Dev..

[bib21] Hirai S., Banba Y., Satake T., Ohno S. (2011). Axon formation in neocortical neurons depends on stage-specific regulation of microtubule stability by the dual leucine zipper kinase-c-Jun *N*-terminal kinase pathway. J. Neurosci..

[bib22] Holahan M.R. (2017). A shift from a pivotal to supporting role for the growth-associated protein (GAP-43) in the coordination of axonal structural and functional plasticity. Front. Cell Neurosci..

[bib23] Honda A., Ito Y., Takahashi-Niki K., Matsushita N., Nozumi M., Tabata H., Takeuchi K., Igarashi M. (2017). Extracellular signals induce glycoprotein M6a clustering of lipid rafts and associated signaling molecules. J. Neurosci..

[bib24] Honda A., Usui H., Sakimura K., Igarashi M. (2017). Rufy3 is an adapter protein for small GTPases that activates a Rac guanine nucleotide exchange factor to control neuronal polarity. J. Biol. Chem..

[bib25] Huang H.D., Lee T.Y., Tzeng S.W., Horng J.T. (2005). KinasePhos: a web tool for identifying protein kinase-specific phosphorylation sites. Nucleic Acids Res..

[bib26] Humphrey S.J., James D.E., Mann M. (2015). Protein phosphorylation: a major switch mechanism for metabolic regulation. Trends Endocrinol. Metab..

[bib27] Humphrey S.J., Azimifar S.B., Mann M. (2015). High-throughput phosphoproteomics reveals in vivo insulin signaling dynamics. Nat. Biotechnol..

[bib28] Huttlin E.L., Jedrychowski M.P., Elias J.E., Goswami T., Rad R., Beausoleil S.A., Villén J., Haas W., Sowa M.E., Gygi S.P. (2010). A tissue-specific atlas of mouse protein phosphorylation and expression. Cell.

[bib29] Igarashi M. (2014). Proteomic identification of the molecular basis of mammalian CNS growth cones. Neurosci. Res..

[bib30] Kuan C.Y., Whitmarsh A.J., Yang D.D., Liao G., Schloemer A.J., Dong C., Bao J., Banasiak K.J., Haddad G.G., Flavell R.A. (2003). A critical role of neural-specific JNK3 for ischemic apoptosis. Proc. Natl. Acad. Sci. USA.

[bib31] Lu Y., Belin S., He Z. (2014). Signaling regulations of neuronal regenerative ability. Curr. Opin. Neurobiol..

[bib32] Lundby A., Secher A., Lage K., Nordsborg N.B., Dmytriyev A., Lundby C., Olsen J.V. (2012). Quantitative maps of protein phosphorylation sites across 14 different rat organs and tissues. Nat. Commun..

[bib33] Lundby A., Andersen M.N., Steffensen A.B., Horn H., Kelstrup C.D., Francavilla C., Jensen L.J., Schmitt N., Thomsen M.B., Olsen J.V. (2013). In vivo phosphoproteomics analysis reveals the cardiac targets of β-adrenergic receptor signaling. Sci. Signal..

[bib34] Margolis F.L., Verhaagen J., Biffo S., Huang F.L., Grillo M. (1991). Regulation of gene expression in the olfactory neuroepithelium: a neurogenetic matrix. Prog. Brain Res..

[bib35] Miller B.R., Press C., Daniels R.W., Sasaki Y., Milbrandt J., DiAntonio A. (2009). A dual leucine kinase-dependent axon self-destruction program promotes Wallerian degeneration. Nat. Neurosci..

[bib36] Navarro X. (2015). Functional evaluation of peripheral nerve regeneration and target reinnervation in animal models: a critical overview. Eur. J. Neurosci..

[bib37] Nix P., Hisamoto N., Matsumoto K., Bastiani M. (2011). Axon regeneration requires coordinate activation of p38 and JNK MAPK pathways. Proc. Natl. Acad. Sci. USA.

[bib38] Nozumi M., Igarashi M. (2017). Vesicular movements in the growth cone. Neurochem. Int..

[bib39] Nozumi M., Nakatsu F., Katoh K., Igarashi M. (2017). Coordinated movement of vesicles and actin bundles during nerve growth revealed by superresolution microscopy. Cell Rep..

[bib40] Nozumi M., Togano T., Takahashi-Niki K., Lu J., Honda A., Taoka M., Shinkawa T., Koga H., Takeuchi K., Isobe T., Igarashi M. (2009). Identification of functional marker proteins in the mammalian growth cone. Proc. Natl. Acad. Sci. USA.

[bib41] Oliva A.A., Atkins C.M., Copenagle L., Banker G.A. (2006). Activated c-Jun *N*-terminal kinase is required for axon formation. J. Neurosci..

[bib42] Oyamatsu H., Koga D., Igarashi M., Shibata M., Ushiki T. (2012). Morphological assessment of early axonal regeneration in end-to-side nerve coaptation models. J. Plast. Surg. Hand Surg..

[bib43] Savastano L.E., Laurito S.R., Fitt M.R., Rasmussen J.A., Gonzalez-Polo V., Patterson S.I. (2014). Sciatic nerve injury: a simple and subtle model for investigating many aspects of nervous damage and recovery. J. Neurosci. Methods.

[bib44] Shin J.E., Geisler S., DiAntonio A. (2014). Dynamic regulation of SCG10 in regenerating axons after injury. Exp. Neurol..

[bib45] Short C.A., Suarez-Zayas E.A., Gomez T.M. (2016). Cell adhesion and invasion mechanisms that guide developing axons. Curr. Opin. Neurobiol..

[bib46] Skene J.H. (1989). Axonal growth-associated proteins. Annu. Rev. Neurosci..

[bib47] Spencer S.A., Schuh S.M., Liu W.S., Willard M.B. (1992). GAP-43, a protein associated with axon growth, is phosphorylated at three sites in cultured neurons and rat brain. J. Biol. Chem..

[bib60] Szklarczyk D., Morris J.H., Cook H., Kuhn M., Wyder S., Simonovic M., Santos A., Doncheva N.T., Roth A., Bork P., Jensen L.J., von Mering C. (2017). The STRING database in 2017: quality-controlled protein-protein association networks, made broadly accessible. Nucleic Acids Res..

[bib48] Tamada A., Igarashi M. (2017). Revealing chiral cell motility by 3D Riesz transform-differential interference contrast microscopy and computational kinematic analysis. Nat. Commun..

[bib49] Tedeschi A., Bradke F. (2013). The DLK signalling pathway: a double-edged sword in neural development and regeneration. EMBO Rep..

[bib50] Villén J., Beausoleil S.A., Gerber S.A., Gygi S.P. (2007). Large-scale phosphorylation analysis of mouse liver. Proc. Natl. Acad. Sci. USA.

[bib51] von Stechow L., Francavilla C., Olsen J.V. (2015). Recent findings and technological advances in phosphoproteomics for cells and tissues. Expert Rev. Proteomics.

[bib52] Wong Y.H., Lee T.Y., Liang H.K., Huang C.M., Yang Y.H., Chu C.H., Huang H.D., Ko M.T., Hwang J.K. (2007). KinasePhos 2.0: a web server for identifying protein kinase-specific phosphorylation sites based on sequences and coupling patterns. Nucleic Acids Res..

[bib53] Yamasaki T., Kawasaki H., Arakawa S., Shimizu K., Shimizu S., Reiner O., Okano H., Nishina S., Azuma N., Penninger J.M. (2011). Stress-activated protein kinase MKK7 regulates axon elongation in the developing cerebral cortex. J. Neurosci..

[bib54] Yamasaki T., Kawasaki H., Nishina H. (2012). Diverse roles of JNK and MKK pathways in the brain. J. Signal. Transduct..

[bib55] Yamasaki T., Deki-Arima N., Kaneko A., Miyamura N., Iwatsuki M., Matsuoka M., Fujimori-Tonou N., Okamoto-Uchida Y., Hirayama J., Marth J.D. (2017). Age-dependent motor dysfunction due to neuron-specific disruption of stress-activated protein kinase MKK7. Sci. Rep..

[bib56] Yan D., Wu Z., Chisholm A.D., Jin Y. (2009). The DLK-1 kinase promotes mRNA stability and local translation in *C. elegans* synapses and axon regeneration. Cell.

[bib57] Yang J., Wu Z., Renier N., Simon D.J., Uryu K., Park D.S., Greer P.A., Tournier C., Davis R.J., Tessier-Lavigne M. (2015). Pathological axonal death through a MAPK cascade that triggers a local energy deficit. Cell.

[bib58] Zhang Z., Cohen D.M. (1996). NaCl but not urea activates p38 and jun kinase in mIMCD3 murine inner medullary cells. Am. J. Physiol..

