# Supplementary material for: Growth Cone Phosphoproteomics Reveals that GAP-43 Phosphorylated by JNK Is a Marker of Axon Growth and Regeneration
Source: iScience. 2018 May 31;4:190–203. doi: 10.1016/j.isci.2018.05.019 (PMC6147025; doi:10.1016/j.isci.2018.05.019)
Supplement: Document S1. Transparent Methods, Figures S1–S7, and Tables S1–S5 [file mmc1.pdf]

ISCI, Volume *4*

Supplemental Information

Growth Cone Phosphoproteomics Reveals that GAP-43 Phosphorylated by JNK Is a Marker of Axon Growth and Regeneration

Asami Kawasaki, Masayasu Okada, Atsushi Tamada, Shujiro Okuda, Motohiro Nozumi, Yasuyuki Ito, Daiki Kobayashi, Tokiwa Yamasaki, Ryo Yokoyama, Takeshi Shibata, Hiroshi Nishina, Yutaka Yoshida, Yukihiko Fujii, Kosei Takeuchi, and Michihiro Igarashi

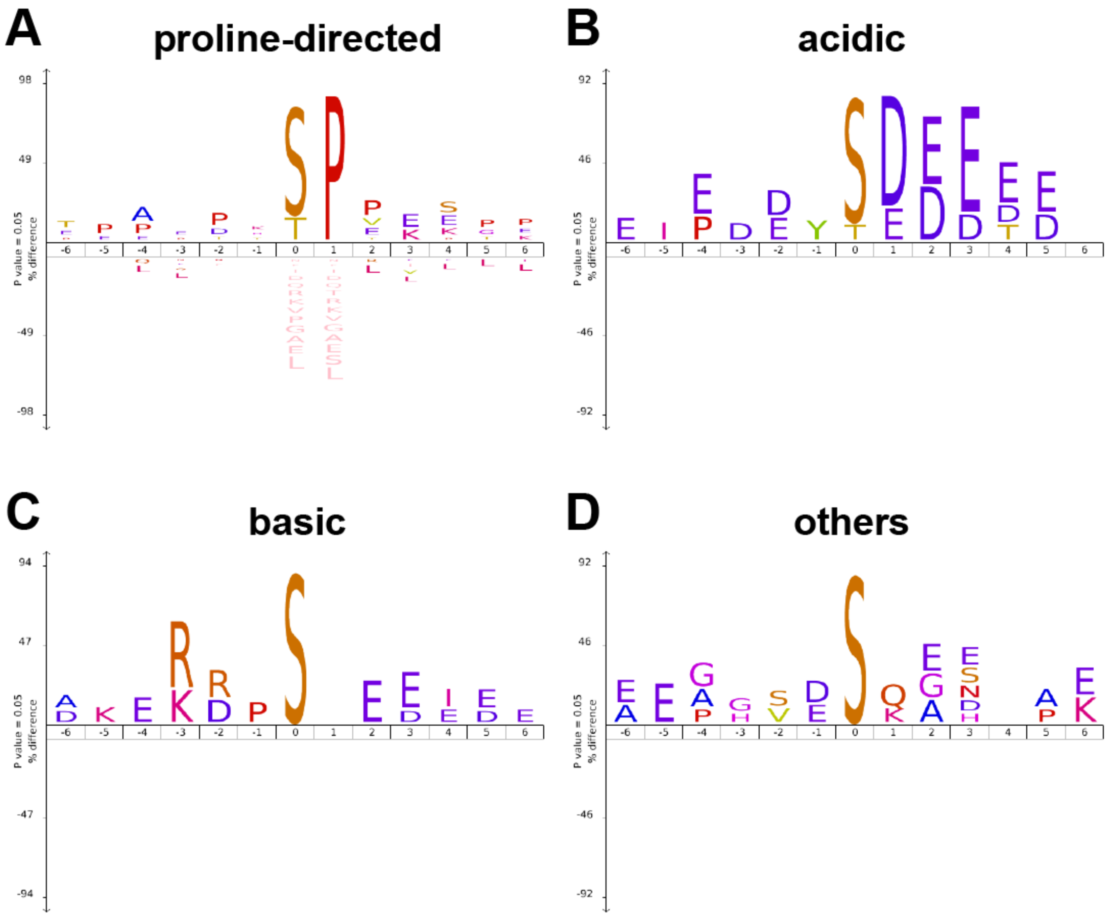

Figure S1, related to Figure 2. Conserved motif sequence pattern for each phosphosite class, corresponding to the classes of protein kinases (Villén et al., 2007) **using the IceLogo web application** (Colaert et al., 2009)**. (A)** P-directed, **(B)** acidic, **(C)** basic, and **(D)** other kinases**.** See also Figure 2A-2B.

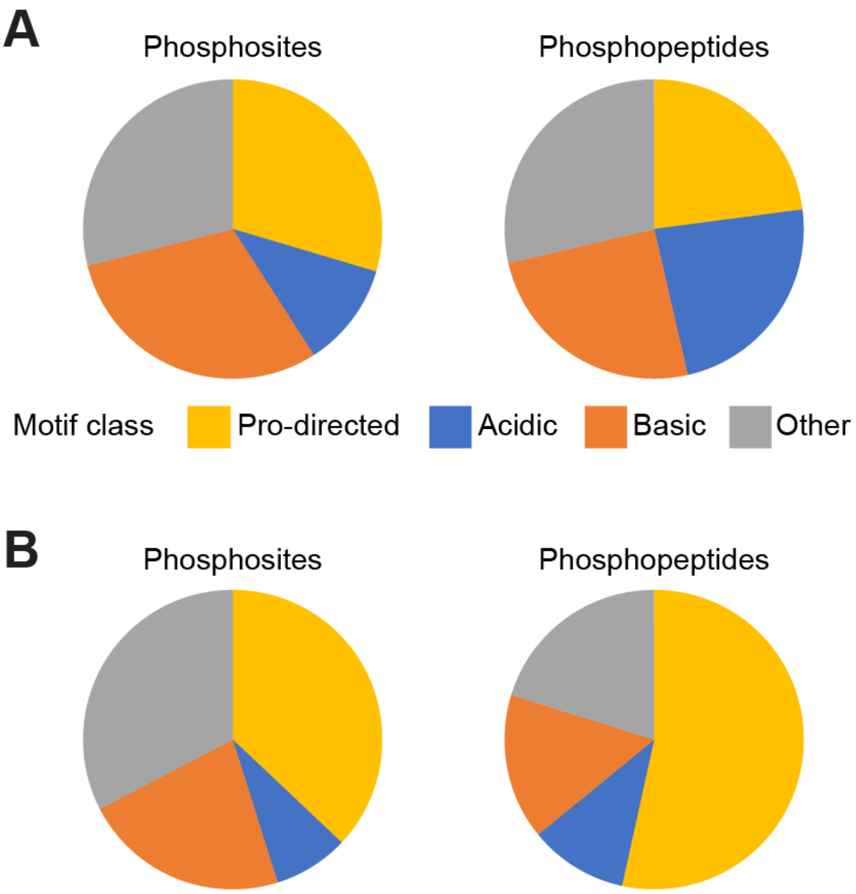

Figure S2, related to Figure 2. “Meta-analysis” of the data appearing in the phopsphoproteomic references (Lundby A et al., 2013 (A); Humphrey et al., 2015b (B)). Although the data showed a tendency for higher P-directed phosphorylation than that reported by (Huttlin et al., 2010), the percentages of the P-directed sites in our paper were higher than those reported in the two papers above (Figure 2A). Each protein kinase group is defined as Villén et al. (2007). See also Figure 2A.

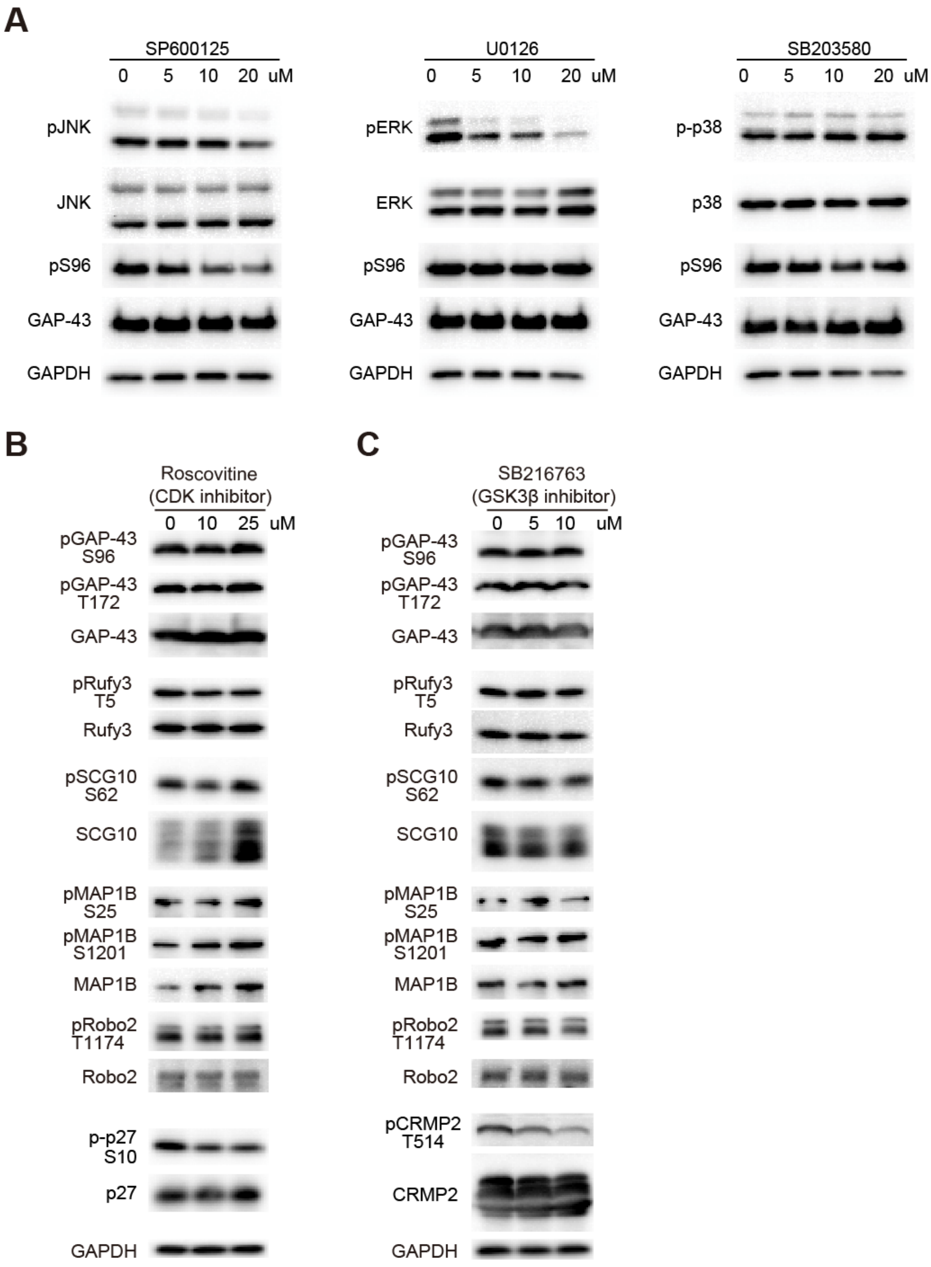

Figure S3, related to Figure 3. **Supplemental results for phosphorylation in cultured mouse neurons in the presence of inhibitors. (A)** Cultured mouse neurons were incubated for 3 h with SP600125, U1026, or SB203580. Each MAPK inhibitor was used at concentrations of 5, 10, or 20 μM. SP600125 inhibited the phosphorylation of GAP-43 at S96 in a dose-dependent manner. No significant effect on the phosphorylation of S96 was observed following incubation with U0126 and SB203580. **(B, C)** CDK **(B)** and GSK3β **(C)** were not responsible for the SP/TP phosphorylated sites of the major GCM phosphoproteins. Cultured mouse neurons were treated with roscovitine (a CDK inhibitor; 10 and 25 μM) or SB216763 (a GSK3 inhibitor; 5 and 10 μM) for 3 h. The inhibitory effect of these inhibitors was evaluated by the phosphorylation levels of p27 (S10) and CRMP2 (T514). As the negative controls, the western blotting results of non-phosphospecific Abs were shown, for all of which the amounts were unchanged in the presence of kinase inhibitors.

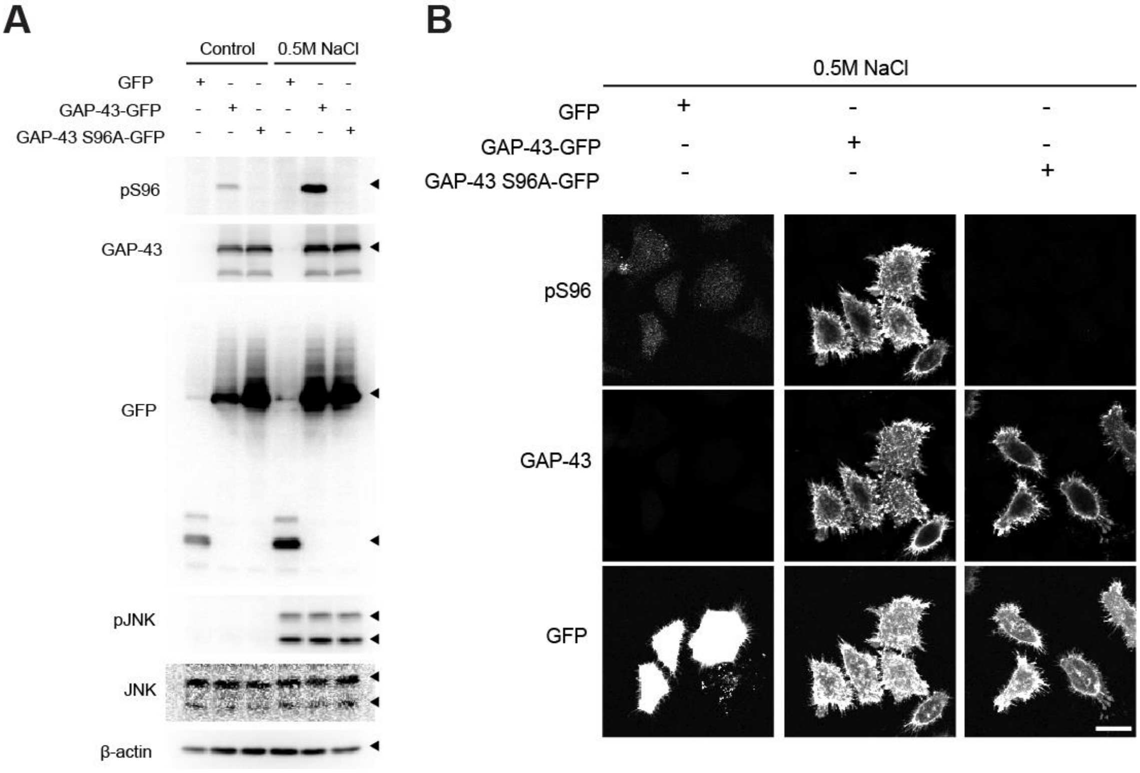

Figure S4, related to Figure 4. Validation of pS96 Ab specificity in transfected HeLa cells (A, B). Extracts from the transfected cells were prepared 30 min after osmotic stress (0.5 M NaCl) for JNK activation and immunoblotted using GAP-43, pS96, GFP, pJNK, JNK, and β-actin Abs (**A**), or the cells were fixed and immunostained with pS96 or pan GAP-43 Abs (**B**).

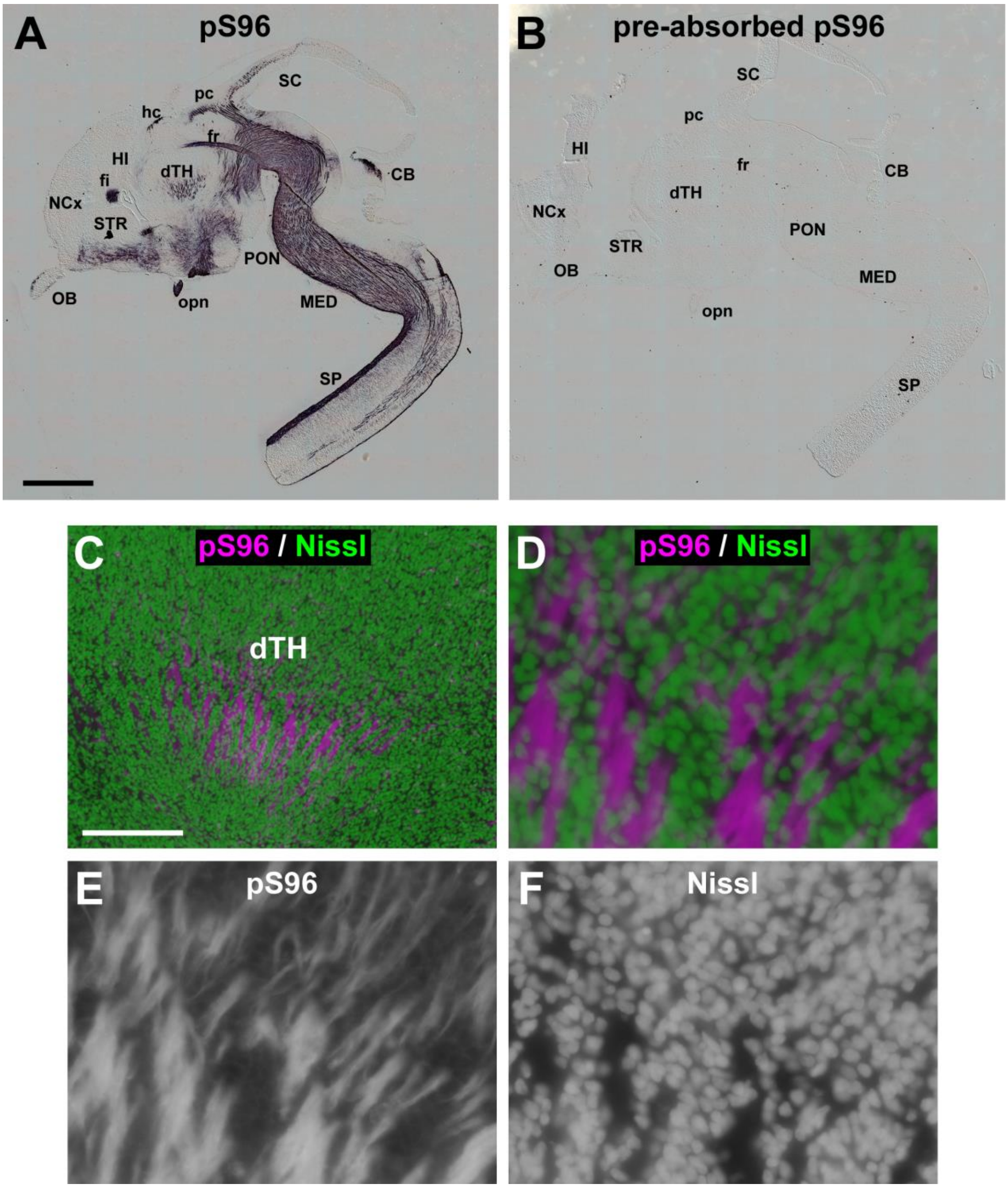

Figure S5, related to Figure 5. Specificity of the antibody against pS96 and its cellular expression pattern *in vivo*. (**A, B**) DIC photomicrographs of adjacent E15 mouse parasagittal brain sections stained with pS96 Ab (**A**) or pS96 Ab preabsorbed with the immunogen peptide (**B**) using the Nickel-enhanced DAB method. Scale bar **(A)**:1 mm (**A, B**). (**C**) Fluorescent immunostaining of pS96 (*magenta*) and fluorescent Nissl staining (*green*) in the dorsal thalamus (dTH) of another section. Scale bar, 200 µm. (**D-F**) High magnification views of (**C**). Note that pS96 is expressed by thalamocortical axons emerging from the dTH, but is absent in their neuronal somata. The scale bar in **(C)** represents 50 µm **(D-F)**. OB, olfactory bulb; NCx, neocortex; STR, striatum; HI, hippocampus, fi, fimbria; opn, optic nerves; fr, fasciculus retroflexus; hc, habenular commissure; pc, posterior commissure; SC, superior colliculus; CB, cerebellum; PON, pons; MED, medulla; SP, spinal cord.

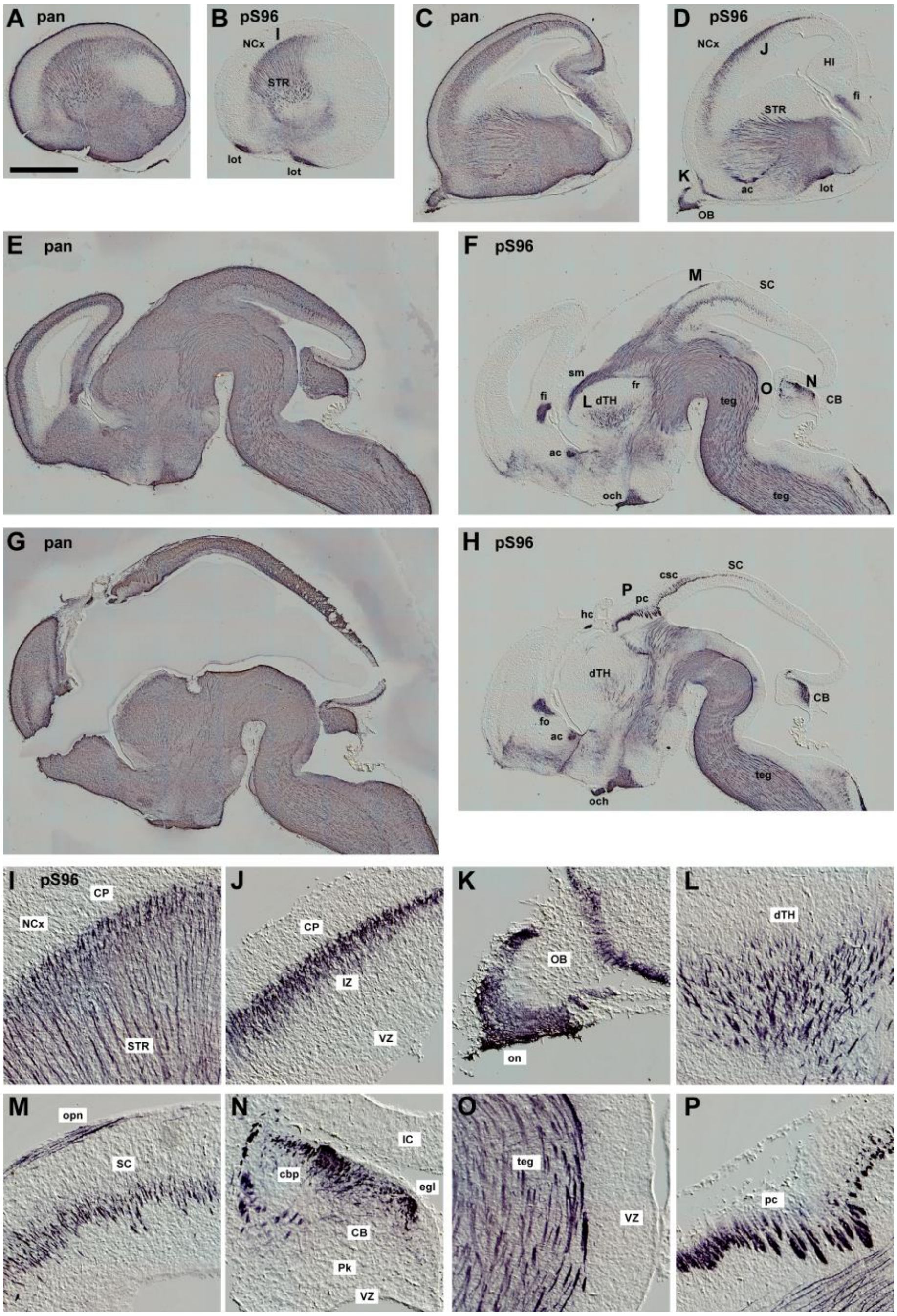

Figure S6, related to Figure 5. **Spatial expression pattern of GAP-43 protein and its pS96 in E15 mouse brain.** (**A - H**) Photographs of parasagittal sections immunostained with pan-GAP-43 (*pan*; **A**, **C**, **E,** and **G**) or pS96 Abs (*pS96*; **B**, **D**, **F,** and **H**). Adjacent sections are displayed in lateral-to-medial order. GAP-43 protein was expressed in most of the differentiated neurons; in contrast, pS96 was confined to the axonal processes and not found in the cell bodies. (**I - P**) Enlarged views of pS96 staining at the regions marked by letters (**(I)** in **(B)**, **(J) - (K)** in **(D)**, **(L) - (O)** in **(F)**, and **(P)** in **(H))**. All pictures are DIC microscopic images of DAB-stained sections. The scale bar shown in **(A)** represents 1 mm (**A – H**), or 200 μm (**I – P**), respectively. lot, lateral olfactory tract; ac, anterior commissure; fo, fornix; och, optic chiasm; sm, stria medullaris; teg, longitudinal tegmental tracts; csc, commissure of the superior colliculus; CP, cortical plate; IZ, intermediate zone; VZ, ventricular zone; on, olfactory nerve; IC, inferior colliculus; cbp, cerebellar peduncles; egl, external granular layer; Pk, Purkinje cell layer. See legend of Figure S5 for other abbreviations.

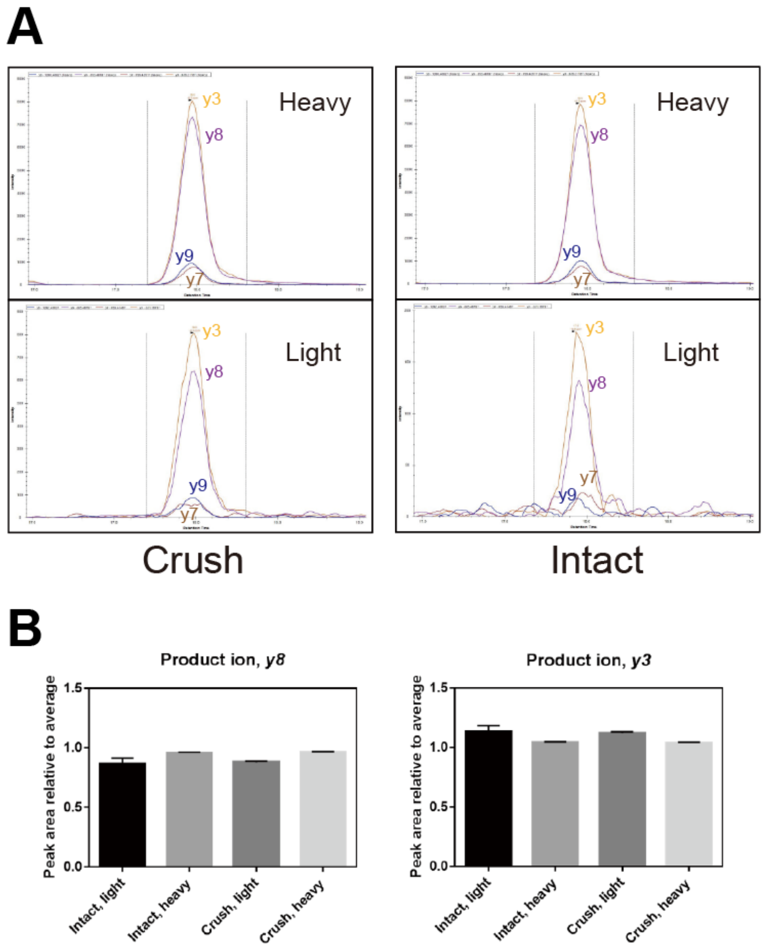

Figure S7, related to Figure 7. Examination of the peptides used for MRM quantitation of GAP-43 pS96 after sciatic nerve injury in adult mice. (A) Representative MRM chromatogram of light and heavy target peptides. MRM transitions were built with the aid of Skyline software (see *Methods*) using the peptide sequence (EGDGSATTDAAPATpSPKAEEPSK). This peptide was phosphorylated at S96 and was found exclusively in the crushed sciatic nerve, according to shotgun proteomic analysis. Note that this peptide contained one missed trypsin cleavage, whereas the pS96-containing peptide without the missed cleavage (EGDGSATTDAAPATpSPK) was not detected in any samples. We therefore chose the former longer peptide as a target peptide for the MRM assay. **(B)** Quantitation of GAP-43-derived peptides phosphorylated at S96. Note that quantitation was most precise when the transitions producing y8 and y3 ions from the target peptide were selected for MRM. The relative area of the product ion chromatograms was in essentially the same range for the entire set of MRM quantitation assays under the same conditions, indicating no significant interference from other contaminating ions.

Table S1, related to Figure 1. Major phosphorylation sites with frequencies more than 100 times in GCM derived from rat P1 brain.

Protein name	Phosphorylation site	Frequency	Kinase group	Protein category
**Gap43**	S96	542	Proline-Directed	Signaling
Ncam1	S784	503	Basic	Cell Adhesion
**Marcksl1**	S22	354	Proline-Directed	Signaling
Mtap1b	S1493	350	Proline-Directed	Cytoskeleton
Stmn1	S25	337	Proline-Directed	Cytoskeleton
Stmn2	S62	287	Proline-Directed	Cytoskeleton
Mtap1b	S1304	280	Proline-Directed	Cytoskeleton
Stmn1	S38	265	Proline-Directed	Cytoskeleton
**Gap43**	T172	245	Proline-Directed	Signaling
Mtap1b	S25	204	Proline-Directed	Cytoskeleton
Mapt	T542	176	Proline-Directed	Cytoskeleton
**Marcks**	S27	171	Proline-Directed	Signaling
Rras2	S186	162	Proline-Directed	Small GTPase
Mtap1b	S1435	148	Proline-Directed	Cytoskeleton
Ncam1	S888	131	Proline-Directed	Cell Adhesion
Dnajc5	S10	126	Basic	Chaperone
Add1	S12	123	Proline-Directed	Cytoskeleton
Marcks	S46	121	Proline-Directed	Signaling
Map2	T1652	119	Proline-Directed	Cytoskeleton
Mtap1b	S1200	110	Proline-Directed	Cytoskeleton
**Marcks**	S29	106	Others	Signaling
Mtap1b	S1464	105	Proline-Directed	Cytoskeleton
**Dpysl3**	S522	100	Proline-Directed	Signaling

The protein name (vertebrate-specific proteins are shown in *bold*), phosphorylation site, phosphopeptide frequency, corresponding protein kinase classification (protein kinase; Villén et al., 2007), and protein classification (see Fig. 1A-1B) are shown.

Table S2, related to Figure 3. Anti-phospho-specific Abs used in this paper.

Protein Name (abbreviated)	Accession number	Phosphorylation site	Sequence of antigen peptide	Amino acid number
GAP-43	NM_017195	S96	DAAPATpSPKAEE	90-101
GAP-43	NM_017195	T172	VTDAAATpTPAAED	165-177
GAP-43	NM_017195	S142	KATTDNpSPSSKA	136-147
Rufy3	NP_001020298	T5	MSALpTPPTDMP	1-11
SCG10	NP_445892	S62	CLILKPPpSPISEA	56-67
MAP1B	NP_062090.1	S25	CNPAATTpSPSLSH	19-30
MAP1B	NP_062090.1	S1201	CASASTIpSPPSSM	1196-1206
Robo2	NP_115289	T1174	CDEDRVPpTPPVRG	1167-1179

All antibodies were produced by Sigma-Aldrich. “pS” and “pT” in the column “Antigen Peptide Sequence” indicate phosphoserine and phosphothreonine, respectively.

Table S3, related to Figure 3. Abs used for immunodetection in this paper.

Antibody	Supplier	Species	Dilution		
			WB	IF	IHC
β-tubulin III (clone TUJ1)	Covance	mouse	-	1:500	-
β-tubulin III Biotinylated (clone TUJ1)	R&D systems	mouse	-	-	1:500
GAP-43	Frontier Institute co., ltd	guinea pig	-	1:500	1:2000 - 1:1000
GAP-43	Millipore	rabbit	1:1000	1:1000	-
GAP-43	Sigma	mouse	1:1000	1:200	-
JNK (#9252)	Cell Signaling Technology	rabbit	1:1000	-	-
p-JNK (#4668)	Cell Signaling Technology	rabbit	1:1000	1:500	-
ERK1/2 (#4695)	Cell Signaling Technology	rabbit	1:1000	-	-
p-ERK1/2 (#4370)	Cell Signaling Technology	rabbit	1:1000	-	-
p38 (#8690)	Cell Signaling Technology	rabbit	1:1000	-	-
p-p38 (#4511)	Cell Signaling Technology	rabbit	1:1000	-	-
JNK2 (#9258)	Cell Signaling Technology	rabbit	1:1000	-	-
JNK3 (#2305)	Cell Signaling Technology	rabbit	1:1000	-	-
JNK1 (clone SC-1648)	Santa Cruz Biotechnology	mouse	1:1000	-	-
MAP-1B (sc-8970)	Santa Cruz Biotechnology	goat	1:1000	-	-
Rufy3 (PA5-31311)	Pierce	rabbit	1:1000	-	-
STMN2 (SCG10) (NBP1-49461)	Novus Biologicals	rabbit	1:1000	1:1000	-
Robo2	Tamada et al., 2008	rabbit	1:1000	-	-
GAPDH	MBL Japan	mouse	1:2000 - 1:1000	-	-
p-GAP-43 (S96) (clone 18-10H-9H)	our group / FUJIFILM Wako	mouse	1:1000	1:2000	1:2000
p-GAP-43 (S96)	our group	rabbit	1:1000	1:100	1:1000
p-GAP-43 (T172)	our group	rabbit	1:1000	-	-
p-Rufy (T5)	our group	rabbit	1:1000	-	-
p-Rufy (T51)	our group	rabbit	1:1000	-	-
p-SCG10 (S62)	our group	rabbit	1:1000	-	-
p-MAP1B (S25)	our group	rabbit	1:1000	-	-
p-MAP1B (S1201)	our group	rabbit	1:1000	-	-
CRMP-2 (#35672)	Cell Signaling Technology	rabbit	1:1000	-	-
p-CRMP-2 (T514) (#9397)	Cell Signaling Technology	rabbit	1:1000	-	-
p27	BD	mouse	1:1000	-	-
p-p27 (S10)	Abcam	rabbit	1:1000	-	-
L1 (clone 324)	Merck	rat	-	-	1:500

WB: Western blotting; IF: immunofluorescence; IH: immunohistochemistry.

Table S4, related to Figure 3. The sequences of siRNAs used in this paper.

siRNA target	Target sequence (5'→3')
MAPK8 (JNK1)	GGAACGAGUUUUAUGAUGA
	GUUAGAUCAUGAAAGAAUG
	UCACUCUGCUGGAAUUAUU
	UUGUUAUCCAAAAUGCUAG
MAPK9 (JNK2)	UCACUGUUCUAAAACGUUA
	CUAGCAACAUUGUAGUAAA
	CUGGUAUCAUUCAUAGAGA
	GCCACCACCUCAAAUUUAU
control (scramble)	UGGUUUACAUGUCGACUAA

Table S5, related to Figure 7. Phosphoproteomics of the injured sciatic nerves in adult mice.

A

**Animal**	**Group**	**Peptide matches**	**Peptide sequence containing S96**	**Phosphorylation**
#127	Crushed	14	EGDGSATTDAAPATSPK<br>EGDGSATTDAAPATSPK<br>EGDGSATTDAAPAT***pS***PKAEEPSK<br>EGDGSATTDAAPAT***pS***PKAEEPSK	 <br>S96<br>S96
#123	Crushed	10	EGDGSATTDAAPATSPK<br>EGDGSATTDAAPATSPK<br>EGDGSATTDAAPAT***pS***PKAEEPSK	 <br>S96
#134	Crushed	9	EGDGSATTDAAPATSPK<br>EGDGSATTDAAPATSPK	
#135	Crushed	4	EGDGSATTDAAPATSPK	
#136	Crushed	6	EGDGSATTDAAPATSPK<br>EGDGSATTDAAPAT***pS***PKAEEPSK	 S96

B

**Target peptide**	**Label**	**Charge**	**m/z**	**Product ion**
EGDGSATTDAAPATpSPKAEEPSK	Light	3	766.332	y9<br>y3<br>y7<br>y8
EGDGSATTDAAPATpSPKAEEPS[^{13}C(6)^{15}N(2)]K	Heavy	3	769.004	y9<br>y3<br>y7<br>y8

(A) The peptides identified containing S96 of GAP-43. The samples from crushed or intact sciatic nerves were separated with SDS-PAGE, and gel slices containing GAP-43 were subjected to in-gel trypsin digestion. Detected phosphorylation of the peptides is shown with italic and bold letters. *Peptide matches*: the number of peptides matched to GAP-43 with a Mascot score larger than the identity threshold and an FDR < 5%. **(B)** MRM transitions for absolute quantitation of GAP-43 phosphorylated at S96. MRM transitions designed for the absolute quantitation of GAP-43 phosphorylated at S96 were generated using Skyline software version 3.1 (University of Washington). y3 and y8 light product ions were sufficiently intense for accurate quantitation, whereas those of the y7 and y9 ions were too weak to define the peak area, in particular in the intact nerve. We therefore selected the former two ions for the above purpose (see also Figure S7).

Legends to the Datasets

Dataset 1. Phosphoproteomics of the GCM proteins. A: Time; B: Precise molecular weight; C: Precise m/z; D: Precise charge (z); E: Protein number (see Dataset 2); F: Best sequence; G: Modification; H: Confidence; I: Theoretical mass; J: Charge (z).

Dataset 2. Identified GCM proteins analyzed with phosphoproteomics. A: Protein number (see Dataset 1); B: Unused; C: Total; D: % coverage; E: Accession number; F: Protein name. *Yellow line*: no phosphorylation-modified peptides were found (only other modifications such as methylation etc.); *Red line:* no phosphopeptides with > 95% reliability were found. They have been deleted from the total counts (Figure 1).

Dataset 3. Enrichment analysis of the GCM phosphoproteins. *Category*, KEGG BRITE categories were used for enrichment analysis. The number of enriched genes was counted in the 4th level of the categories. If the number of genes included in a category is lower than four, the category is removed from the calculation. *P-value*, P-value of Fisher's exact test; *Q-value*, FDR adjusted P-value; *Genes*, the genes encoding the identified phosphoproteins in GCM. See also Figure 2D.

Transparent Methods

Animals. All the animal experiments were conducted in compliance with the protocol which was reviewed by the Institutional Animal Care and Use Committee and approved by the President of Niigata University (Permit Number: #26 Niigata Univ. Res.74-2). Postnatal SD rats (Nihon-SLC, Shizuoka, Japan) were used for GCM preparation. Timed-pregnant ICR mice (Nihon-SLC, Shizuoka, Japan) were used for developmental immunohistochemical analysis. Noon of the day on which the plug was detected was designated as E0.5. Brains were removed from the animals that had been deeply anesthetized with sodium pentobarbitone (Nembutal, Abbott, North Chicago, IL, USA; 50 mg/kg of body weight). C57BL/6NCrl mice (Charles River Laboratories Japan, Yokohama, Japan) were used for adult nerve regeneration study (See below).

Phosphoproteomics analysis of the GCM fraction. GCM prepared from P1 rat forebrains, its validation, protein extraction, and protein digestions were performed as described previously (Ellis et al., 1985; Gordon-Weeks, 1988; Nozumi et al., 2009). The protein extract (2 mg) from the GCM lysate was suspended in 8 M urea containing 50 mM triethylammonium bicarbonate (TEAB) (pH 8.0). The proteins were reduced by the addition of 50 mM tris(2-carboxyethyl)phosphine and incubated for 2 h at 37°C; the sample was then cooled to room temperature prior to the addition of 20 mM methylmethanethiosulfonate and the cysteines were alkylated for 15 min. The protein mixture was diluted with 50 mM TEAB to a final urea concentration of 1.6 M, then digested by adding 20 μg trypsin (Sciex) and incubating for 16 h at 37°C. The sample was desalted using a Sep-Pak C18 cartridge (Waters Corporation), according to the manufacturer's instructions.

The phosphopeptide mixture was fractionated into six fractions on a strong cation exchange chromatography cartridge (Sciex), using a stepwise gradient of KCl (0, 20, 50, 100, 175, and 350 mM). Each resulting fraction was desalted, solubilized in 0.1% formic acid, and analyzed using a nanoLC-QSTAR Elite mass spectrometer (Sciex) with a NanoSpray III source. Ion source conditions were "ionspray voltage" = 1800 V; "curtain gas" = 20; "declustering potential 1" = 60 V; "focusing potential" = 250 V; and "declustering potential 2" = 15 V. Separation by nanoLC (KYA Technologies) was performed at a constant flow rate of 200 nl/min with a 190-min gradient. A QSTAR Elite mass spectrometer was used in standard MS/MS data-dependent acquisition mode. Survey MS spectra (0.5-sec) were collected (m/z 400-1800), followed by three MS/MS measurements of the most intense parent ions (20 counts/sec threshold, 2-5 charge state, m/z 65-2000 mass range for MS/MS), using the manufacturer's "smart exit" setting 2. Previously targeted parent ions were excluded from repetitive MS/MS acquisition for 60 sec (50 mDa mass tolerance).

All searches were performed against rodent CDS FASTA and annotated with the PANTHER Classification System information. An FDR calculation was performed as described previously (Tang et al., 2008). Phosphopeptide enrichment by IMAC with PHOS Select Iron Affinity Gel (Sigma-Aldrich) was performed essentially as described (Kokubu et al., 2005; Villén and Gygi, 2008). Data files were processed with ProteinPilot 2.0 (Sciex) using the Paragon algorithm (Shilov et al., 2007).

Bioinformatic analyses of the phosphoproteomic data. Serine and threonine residues in the phosphopeptides detected by the phosphoproteomics analysis were identified as phosphorylation sites (phosphosites). The counts of the phosphopeptides by the spectroscopy were used as an index that represents the abundance of phosphorylation. Phosphoproteins containing the detected phosphopeptides were classified into 14 functional protein categories. Phosphosites weighted by their counts were similarly classified. The phosphosites were further divided into P-directed and non–P-directed phosphorylation events. Kinases responsible for the identified phosphopeptides were predicted using KinasePhos server (Wong et al., 2007) with 100% specificity. Conserved motif sequence patterns for each phosphosite class were generated with the iceLogo web application (Colaert et al., 2009). The parameters were used as default settings (see Figure S1).

To construct a molecular interaction network (enrichment analysis), KEGG BRITE categories (Kanehisa et al., 2017) were used for enrichment analysis. The number of enriched genes

was counted in the 4th level of the categories. If the number of genes included in a category is lower than four, the category is removed from the calculation. The STMN2-4 and DBN1 genes, which are often observed in the category of cytoskeletal modifiers, were not assigned to KEGG Orthology annotations in the version on KEGG BRITE used in this study. Thus, the annotation was manually assigned for these genes prior to performing enrichment analysis. Namely, the protein–protein association data of the STRING database (Szklarczyk et al., 2017) were imported into Cytoscape (Smoot et al., 2011) using the stringApp plugin. See also Figure 2D and Dataset 3.

For "metanalysis" of the high-throughput phosphoproteomics analyses performed by other groups (Lundby et al., 2013; Humphrey et al., 2015b), those phosphoproteomic data were downloaded and re-analyzed. The detected phophosites were classified into four motif classes, including proline-directed, acidic, basic, and others, according to the following criteria: (1) P at +1 (P), (2) five or more E/D at +1 to +6 (A), (3) R/K at -3 (B), (4) D/E at +1/+2 or +3 (A), (5) two or more R/K at -6 to -1 (B), (6) others (O). The intensities of the phosphosites were added to calculate the fraction. In addition, the intensity value was normalized and the phosphosites with a score ≥ 20 or more were extracted for high intensity phosphosites. The fraction of the high intensity groups was calculated as the sum of the original intensity. See Figure S2.

Western blotting for cells and mouse brains. The newly generated polyclonal Abs were produced by Sigma-Aldrich, using phosphopeptides as antigens in rabbits (listed in Table S2). The usages of other Abs were summarized in Table S3. Western blotting procedures were described previously (Adachi et al., 2014). The cultured neurons were lysed with TNE buffer (20 mM Tris-HCl [pH 7.5], 150 mM NaCl, 1 mM EDTA, 1 mM phenylmethylsulfonyl fluoride, 1 mM NaF, 1 mM Na_3VO_4, 10 μg/ml pepstatin, 10 μg/ml leupeptin, 1% Triton X-100). Experiments using inhibitors of the protein kinases were performed as described previously (Kumar et al., 1999).

Neuronal culture, RNAi, and pharmacological studies. Mouse cortical neurons on E15 were dissociated and cultured as described previously (Nozumi et al., 2009). For RNAi experiments, the siRNA sequences (Accell SMARTpool, Dharmacon) used were as follows (Table S4): mouse JNK1 (#E-040128-00) and JNK2 (#E-040134-00). Knock-down efficiency for each siRNA was evaluated by immunoblot analysis 48 h after transfection. Accell Red Non-targeting siRNA (#D-001960-01) was used as a negative control.

DNA Transfection. An expression plasmid was constructed for fused protein of rat GAP-43 and EGFP under the control of the CAG promoter. Then, serine-96 was replaced with alanine by KOD-Plus-Mutagenesis Kit (Toyobo). Hela cells were transfected with these plasmids by polyethyleneimine.

Immunohistochemistry. Brains were fixed by immersion in 0.12 M phosphate buffer (PB; pH 7.4) with 4% paraformaldehyde (PFA) for 2-3 days, cryoprotected in 0.1 M PB with 30% sucrose for additional 2-3 days, and embedded in OCT compound (Sakura Finetechnical Co., Ltd., Tokyo, Japan). Coronal sections were cut at a thickness of 20 μm and thaw-mounted on glass slides. Primary Abs used for immunohistochemistry included mouse anti-pS96 (1/2000 dilution) that was biotinylated with Biotin Labeling Kit-NH_2 (Dojindo Laboratories, Kumamoto, Japan), guinea pig anti-pan-GAP-43 (GAP43-GP-Af500, Frontier Institute Co., Ltd., Ishikari, Japan, 1/2000 - 1/1000 dilution) and rat anti-L1 (clone 324, Merck, Darmstadt, Germany, 1/500 dilution). Sections were immunohistochemically stained as previously described (Tamada et al., 2008) with some modifications. All procedures were performed at room temperature. Single immunostaining was performed using a standard avidin–biotin complex (ABC) method (Vectastain ABC Elite kit, Vector Laboratories, Burlingame, CA, USA). Sections were incubated with methanol containing 0.3% H_2O_2 for 30 min, washed three times for 10 min each with phosphate buffer saline (PBS) containing 0.2% Triton X-100 (PBST), blocked with 10% normal goat serum (NGS) in PBST for 30 min, and incubated overnight with primary antibodies diluted in NGS-PBST. The next day, sections were washed three times with PBST, reacted with 1/400 biotinylated goat anti-guinea pig IgG (BA7000, Vector) in NGS-PBST for 1 h in the case of pan GAP-43, washed three times with PBST and twice

with PBS, incubated with 1/400 elite ABC in PBS for 1 h, and washed three times with PBS. Color was developed using the nickel-enhanced diaminobenzidine (DAB) method (0.02% DAB, 0.003% H_2O_2, 2.5% $NiSO_4$ in 0.1 M acetate buffer, pH6.0). For multiple fluorescent staining, sections were incubated with the primary antibodies and then with Cy5- or Alexa Fluor 488–conjugated secondary antibodies or Cy3-conjugated streptavidin (all from Jackson ImmunoResearch Inc., West Grove, PA, USA). In some cases, sections were further stained for nuclear DNA with 4', 6-diamidino-2-phenylindole (DAPI; Thermo Fisher Scientific Inc., Waltham, MA, USA) or Nissl-stained with NeuroTrace 500/525 (Molecular Probes, Eugene, OR, USA). DAB-stained samples were observed through an upright microscope (BX63, Olympus, Tokyo, Japan) equipped with differential interference contrast (DIC) optics. Multi-field images were acquired with a CCD camera (DP72, Olympus) and stitched with cellSens software (Olympus). Fluorescence images were acquired with a confocal microscope (FV3000, Olympus) or the upright microscope.

Immunocytochemistry. Cultured neurons were fixed with 4% PFA for 15 min at 37°C, permeabilized with PBS containing 0.1% Triton X-100 and 5% BSA for 1hr. Cells were immunostained with the primary and secondary antibodies. For visualization of F-actin, cells were incubated with Rhodamine-phalloidin (Sigma-Aldrich) for 1 h. Fluorescence images were acquired with a confocal laser scanning microscope (FV1200, Olympus).

Sciatic nerve injury. Following a standard protocol (Savastano et al., 2014; Shin et al., 2014), male C57BL/6NCrl mice at 9 weeks of age or older were anesthetized with a cocktail of ketamine and xylazine, and the nerve was crushed with fine forceps (Fontax, INOX #5) for 30 s. The mice were sacrificed on 1, 3, or, 7 days after the operation.

For the immunofluorescence study of the regenerating axons, the mice were perfused intracardially with PBS followed by 4% PFA. Then, the nerves were washed with PBS, immersed in 0.1 M PB with 20% sucrose for an additional 1-2 day, and cut into 20-μm-thick longitudinal sections. The nerve samples were immunostained as described previously (Shin et al., 2012, 2014). Alexa Fluor 488-conjugated Abs or Alexa 594-conjugated streptavidin (Jackson ImmunoResearch Inc.) were used as the secondary Abs. 3D Multiple Fluorescence images were taken with a confocal microscope (FV1200, Olympus) using 10× air objective along the nerve.

Regeneration was evaluated by the regeneration index (Shin et al., 2012; 2014). With ImageJ and MATLAB (Mathworks, USA), 3D stack images were projected along the two axes perpendicular to the nerve and then moving-averaged along the nerve axis to obtain the 1D signal decay. The index was calculated as the distance from the injury site to the point where the signal decays by half.

For western blotting of the sciatic nerves, they were dissected by 1-mm length (see Figure 7A). The samples were homogenized using an ultra-sonicator in sample buffer (125 mM Tris-HCl pH 6.8, 20% glycerol and 4% SDS) with protease inhibitors (10μg/ml leupepsin, 10μg/ml pepstatin, 0.02mM p-APMSF and 1mM EDTA) and phosphatase inhibitors (1.0mM NaF, 1.15mM Na_2MoO_4 and 1.0mM Na_3VO_4). Protein bands were visualized using an ECL Prime kit (GE health care life science, Piscataway, USA). The contralateral intact samples were also used as the control. The relative protein levels recognized by the pan-GAP-43 and pS96 Abs were calculated as the ratio of Crush/Intact nerves.

MS of regenerating axons. The protein extract from the injury site or the mock-operated site of the sciatic nerve was prepared as described above. Five equal aliquots of protein extract (19 μg) were separated in parallel on a polyacrylamide gel (ANK KD, Mini-PROTEAN TGX; Bio-Rad Laboratories) and stained with Coomassie Brilliant Blue R-250. The five gel portions containing GAP-43, as indicated by immunoreactivity with anti–pan-GAP-43 Ab, were manually excised and collected in a low-binding microcentrifuge tube for in-gel trypsin digestion. Protein identification was carried out using Mascot version 2.2.1 (Matrix Science).

The gel slices were reduced with 10 mM dithiothreitol, carbamide methylated with 55 mM iodoacetamide, and subjected to in-gel trypsin digestion essentially as described previously (Katayama et al., 2001) to improve recovery of the digested peptides. The peptides from each sample

were finally dissolved in 15 μl of 0.2% trifluoroacetic acid and assayed with the BCA method modified for peptide assays (Kappoor et al., 2009). Each sample (5 μl, 0.5-0.8 μg) was injected into a nano-flow-LC (Eksigent nanoLC 415 with ekspert cHiPLC; Sciex) coupled with a tandem MS (TripleTOF5600; Sciex). Analysis was conducted in duplicate for each sample under trap and elute mode using a ChromeXP C_{18} Chip column (200 μm × 0.5 mm) as a trap column, and the same column (75 μm × 150 mm) as the analytical one. Mobile phases A and B were 0.1% formic acid and 0.1% formic acid in acetonitrile, respectively. Peptides were eluted using 30-min gradients from 2% to 32% B at 300 nl/min. MS spectra (250 msec) followed by 10 MS/MS spectra (100 msec each) were acquired in data-dependent mode. The dynamic exclusion time was set at 12 sec. Autocalibration using 50 fmol of tryptic peptides of bovine serum albumin was performed every five to nine samples. Protein identification was carried out using Mascot version 2.2.1 (Matrix Science) as a search engine. The raw data generated by Analyst TF 1.6 (Build 6211) were converted to Mascot generic files by MS Converter (Sciex) and searched against an in-house constructed UniProtKB mouse reference proteome database (49,878 sequences, 29 May 2015 release) using the instrument settings for the ESI-QUAD-TOF spectrometer. The peptide and MS/MS tolerance were set at ± 20 ppm and ± 0.1 Da, respectively. Modification settings were: fixed modification, carbamidomethylation of cysteine, variable modifications, deamidation of asparagine and/or glutamine, phosphorylation of serine and/or threonine, *N*-terminal glutamine to pyroglutamate, *N*-terminal glutamate to pyroglutamate, and oxidation of methionine. A maximum of two missed cleavages was allowed. The significance threshold was set at $p < 0.05$, which gave an FDR of < 0.05 for all identification results. Only peptides with a score exceeding the "Identity threshold" were employed. "Require bold red" was checked to avoid redundancy in protein identification. Quantitation of GAP-43 was performed using the normalized spectral abundance factor (NSAF) (Paoletti et al., 2006). The spectral abundance factor (SAF) was first calculated by dividing the number of spectral counts for each protein by the protein mass or protein length. SAF values were then normalized by dividing by the sum of all SAFs for proteins in a sample to give NSAFs. The schematic procedure is shown in Figure 7A.

HR-MRM assay for absolute quantitation of pS96 GAP-43 in the crushed nerve. For HR-MRM, the light (EGDGSATTDAAPATpSPKAEEPSK; m/z = 766.3321) and heavy (EGDGSATTDAAPATpSPKAEEPS [13C(6)15N(2)]K; m/z = 769.0035) peptides were used; the latter was labeled with stable isotopes (SIs; Narumi et al., 2012; Adachi et al., 2016). The internal standard peptide was uniformly labeled with ^{13}C and ^{15}N at the carboxyl-terminal lysine (AQUA Peptides, Sigma-Aldrich Life Science). MRM transitions were generated by Skyline software version 3.1 (University of Washington, Seattle, WA, USA). The MRM assay was performed with a TripleTOF 5600+ MS in HR-MRM mode. LC–tandem MS was performed similarly to shotgun analysis.

MRM transitions were built with the aid of Skyline software (University of Washington) using the above light sequence peptide. The peptide contained one missed cleavage by trypsin, while the peptide phosphorylated at S96 without a missed cleavage (EGDGSATTDAAPATpSPK) were not observed in any samples (Table S5A). Among the 11 peptides matching the corresponding sequence of GAP-43 for five mice, six peptides were shorter and lacked pS96, whereas the other five peptides were longer due to a missed cleavage and contained pS96. None of the shorter peptides contained pS96 (Table S5A). We therefore chose the former longer peptide as a target peptide for the MRM assay.

MRM transitions designed for absolute quantitation of GAP-43 phosphorylated at S96 was generated using Skyline software version 3.1 (Table S5B). Among four transitions selected for light and heavy target peptides, the y3 and y8 product ions of the light peptides were sufficiently strong for accurate quantitation, while those of y7 and y9 ions were too weak to define a peak area, especially in samples from intact nerve (Figure S6A). We therefore selected y3 and y8 ions for calculation of absolute amounts of phosphorylated GAP-43.

To confirm the lack of interference from contaminants in the MRM-HR assay, the peak areas of y3 and y8 relative to their averaged values were compared among all the samples (see Figure 7; Figure S6; Table S5A-S5B). No significant difference between samples was observed,

indicating that there was no significant interference from contaminants in the assay as well as the accuracy of the MRM-HR assay (Figure S6B).

Statistics. GraphPad Prism (GraphPad Software) was used for statistical analysis and drawing graphs. Biochemical and culture experiments were analyzed using a Student's *t*-test or one-way *ANOVA* with Bonferroni post-hoc tests. The *p*-value for statistical significance was defined as $p < 0.05$. All data are shown as the mean ± standard deviation (SD) or mean ± standard error of the mean (SEM).

Supplemental references

Adachi, J., Narumi, R., and Tomonaga, T. (2016). Targeted phosphoproteome analysis using selected/multiple reaction monitoring (SRM/MRM). Methods Mol Biol *1394*, 87-100.

Adachi, M., Kawasaki, A., Nojima, H., Nishida, E., and Tsukita, S. (2014). Involvement of IQGAP family proteins in the regulation of mammalian cell cytokinesis. Genes Cells *19*, 803-820.

Colaert, N., Helsens, K., Martens, L., Vandekerckhove, J., and Gevaert, K. (2009) Improved visualization of protein consensus sequences by iceLogo. Nat Methods *6*, 786-787.

Gordon-Weeks, P.R. (1988). The ultrastructure of the neuronal growth cone: new insights from subcellular fractionation and rapid freezing studies. Electron Microsc Rev *1*, 201-219.

Kanehisa, M., Furumichi, M., Tanabe, M., Sato, Y., and Morishima, K. (2017). KEGG: new perspectives on genomes, pathways, diseases and drugs. Nucleic Acids Res. 45: D353-D361.

Kappoor, K.N., Barry, D.T., Rees, R.C., Dodi, I.A., McArdle, S.E., Creaser, C.S., and Bonner, P.L. (2009). Estimation of peptide concentration by a modified bicinchoninic acid assay. Anal Biochem *393*, 138-140.

Katayama, H., Nagasu, T., and Oda, Y. (2001). Improvement of in-gel digestion protocol for peptide mass fingerprinting by matrix-assisted laser desorption/ionization time-of-flight mass spectrometry. Rapid Commun Mass Spectrom *15*, 1416-1421.

Kumar S, Jiang MS, Adams JL, Lee JC (1999). Pyridinylimidazole compound SB 203580 inhibits the activity but not the activation of p38 mitogen-activated protein kinase. Biochem Biophys Res Commun *263*, 825-831.

Kokubu, M., Ishihama, Y., Sato, T., Nagasu, T., and Oda, Y. (2005). Specificity of immobilized metal affinity-based IMAC/C18 tip enrichment of phosphopeptides for protein phosphorylation analysis. Anal Chem *77*, 5144-5154.

Narumi, R., Murakami, T., Kuga, T., Adachi, J., Shiromizu, T., Muraoka, S., Kume, H., Kodera, Y., Matsumoto, M., Nakayama, K., Miyamot,o Y., Ishitobi, M., Inaji, H., Kato, K, and Tomonaga, T. (2012). A strategy for large-scale phosphoproteomics and SRM-based validation of human breast cancer tissue samples. J Proteome Res *11*, 5311-5322.

Paoletti, A.C., Parmely, T.J., Tomomori-Sato, C., Sato, S., Zhu, D., Conaway, R.C., Conaway, J.W., Florens, L., and Washburn, M.P. (2006). Quantitative proteomic analysis of distinct mammalian mediator complexes using normalized spectral abundance factors. Proc Natl Acad Sci USA *103*, 18928-18933.

Shilov, I.V., Seymour, S.L., Patel, A.A., Loboda, A., Tang, W.H., Keating, S.P., Hunter, C.L., Nuwaysir, L.M., and Schaeffer, D.A. (2007). The Paragon Algorithm, a next generation search engine that uses sequence temperature values and feature probabilities to identify peptides from tandem mass spectra. Mol Cell Proteomics *6*, 1638–1655.

Shin, J.E., Cho, Y., Beirowski, B., Milbrandt, J., Cavalli, V., and DiAntonio, A. (2012) Dual leucine zipper kinase is required for retrograde injury signaling and axonal regeneration. Neuron *74*, 1015-1022.

Smoot, M.E., Ono, K., Ruscheinski, J., Wang, P.L., and Ideker, T. (2011). Cytoscape 2.8: new features for data integration and network visualization. Bioinformatics *27*, 431-432.

Tamada, A., Kumada, T., Zhu, Y, Matsumoto, T, Hatanaka, Y., Muguruma, K., Chen, Z., Tanabe, Y., Torigoe M., Yamauchi, K., Oyama, H., Nishida, K., and Murakami, F. (2008). Crucial roles of Robo proteins in midline crossing of cerebellofugal axons and lack of their up-regulation after midline crossing. Neural Dev *3*, 29.

Tang WH, Shilov IV, and Seymour SL. (2008). Nonlinear fitting method for determining local false discovery rates from decoy database searches. J Proteome Res *7*, 3661-3667.

Villén J, and Gygi SP. (2008).The SCX/IMAC enrichment approach for global phosphorylation analysis by mass spectrometry. Nat Protocols *3*, 1630-1638.
